# SREBP1-dependent *de novo* fatty acid synthesis gene expression is elevated in malignant melanoma and represents a cellular survival trait

**DOI:** 10.1038/s41598-019-46594-x

**Published:** 2019-07-17

**Authors:** Su Wu, Anders M. Näär

**Affiliations:** 1 Massachusetts General Hospital Center for Cancer Research, Charlestown, MA USA; 2 Department of Cell Biology, Harvard Medical School, Boston, MA USA; 3 Present Address: Department of Biological Chemistry and Molecular Pharmacology, Harvard Medical School, Boston, MA USA; 4 Present Address: Department of Nutritional Sciences & Toxicology, University of California, Berkeley, Berkeley, CA USA

**Keywords:** Melanoma, Computational biology and bioinformatics

## Abstract

*de novo* fatty acid biosynthesis (DNFA) is a hallmark adaptation of many cancers that supports survival, proliferation, and metastasis. Here we elucidate previously unexplored aspects of transcription regulation and clinical relevance of DNFA in cancers. We show that elevated expression of DNFA genes is characteristic of many tumor types and correlates with poor prognosis, especially in melanomas. Elevated DNFA gene expression depends on the SREBP1 transcription factor in multiple melanoma cell lines. SREBP1 predominantly binds to the transcription start sites of DNFA genes, regulating their expression by recruiting RNA polymerase II to promoters for productive transcription elongation. We find that SREBP1-regulated DNFA represents a survival trait in melanoma cells, regardless of proliferative state and oncogenic mutation status. Indeed, malignant melanoma cells exhibit elevated DNFA gene expression after the BRAF/MEK signaling pathway is blocked (e.g. by BRAF inhibitors), and DNFA expression remains higher in melanoma cells resistant to vemurafenib treatment than in untreated cells. Accordingly, DNFA pathway inhibition, whether by direct targeting of SREBP1 with antisense oligonucleotides, or through combinatorial effects of multiple DNFA enzyme inhibitors, exerts potent cytotoxic effects on both BRAFi-sensitive and -resistant melanoma cells. Altogether, these results implicate SREBP1 and DNFA enzymes as enticing therapeutic targets in melanomas.

## Introduction

Cancer cells characteristically achieve hallmark traits that facilitate proliferation, survival, and metastasis^1–3^. One hallmark adaptation is *de novo* fatty acid synthesis (DNFA), metabolic conversion of carbohydrates into lipids *via* acetyl-CoA and NADPH with the aid of multiple lipogenic enzymes, including ATP citrate lyase (ACLY), acyl-coenzyme A synthetase 2 (ACSS2), acetyl-CoA carboxylase (ACACA), fatty acid synthase (FASN), and stearoyl-CoA desaturase (SCD)^4^. DNFA occurs in cancer cells and certain types of healthy cells^5^. In hepatocytes, DNFA activity is primarily regulated at the transcriptional level of mRNAs encoding DNFA enzymes^6^, in response to dietary lipids (e.g. polyunsaturated fatty acids^7–9^) and hormonal cues such as insulin^10^. DNFA also increases during normal embryonic development and adipogenesis to satisfy elevated lipid demands during cell proliferation and fat storage processes, respectively^11,12^.

The transcription factor sterol regulatory element-binding protein 1 (SREBP1) plays a central role in controlling DNFA gene expression, and, by extension, cellular FA/lipid production^13,14^. There are two major mechanisms involved in SREBP1 regulation: mRNA expression and proteolytic processing^15^. The *SREBF1* gene encodes a SREBP1 precursor protein embedded in the endoplasmic reticulum membrane through two transmembrane domains^16–18^. In response to depletion of cellular and membrane lipids, its nuclear form (nSREBP1) is released by site 1 and site 2 proteases^19–21^, translocates into the nucleus and binds to target gene promoters. nSREBP1 activates the transcription of DNFA genes, in concert with other transcription factors such as LXR^22^, USF1^23^, NFY1^24^ and SP1^25^, and co-activators including MED15^26^ and CREBBP^27^. nSREBP1 also participates in activation of *SREBF1* mRNA expression by binding to its own promoter^28^, thus the levels of DNFA mRNAs parallel the changes in *SREBF1* expression^13^.

Elevated DNFA has been demonstrated in many tumor types^29^. Prevailing thought holds that hallmark traits, such as DNFA, emerge via pro-survival signaling pathways driven by oncogene and tumor suppressor alterations^30–33^. Supposed tumor cell dependence on a single oncogenic driver or pathway to sustain proliferation and/or survival has guided the development of targeted cancer therapies^34,35^. However, in clinical settings, tumors harbor highly diverse genetic alterations and exhibit stochastic evolution^36^, which often limits the prognostic and therapeutic value of that supposition^37–40^. Resistance to targeted therapies related to reactivation or bypass of downstream signaling pathways is common^41^. It is unclear whether oncogene alterations maintain hallmark traits such as DNFA in malignant tumors. Furthermore, potential interaction between oncogenic drivers and DNFA has not been fully investigated, especially under the selective pressure of targeted therapies.

We show here that elevated expression of key DNFA enzymes such as SCD is associated with poor prognosis in cancers, including melanomas. We demonstrate the molecular mechanism by which SREBP1 controls DNFA gene transcription in melanoma cells, revealing a regulatory role for RNA polymerase II pause/release. Our cellular analyses further reveal crucial roles for elevated DNFA gene expression in cell proliferation and survival, regardless of whether they are sensitive or resistant to targeted therapies (e.g., BRAF inhibitors).

## Results

### Expression and prognostic value of DNFA genes in cancers

**Figure 1:**
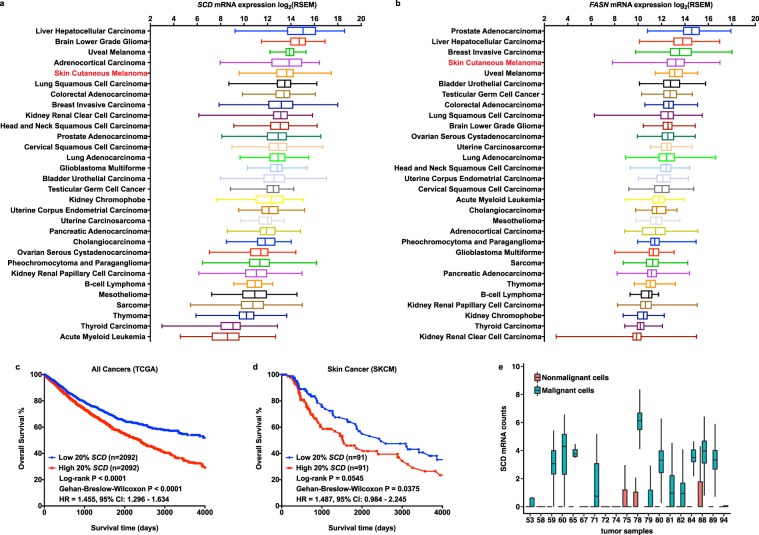
Elevated expression of DNFA genes is prevalent in many cancers, including melanomas, and has prognostic value. (**a**,**b**) Expression of *SCD* and *FASN* genes was compared using RSEM normalized RNA-Seq data from 10,210 tumor samples downloaded from The Cancer Genome Atlas (TCGA). The box and whisker plots represent gene expression in 30 TCGA cancer types. (**c**,**d**) We divided patients into two groups based on the ranking of *SCD* mRNA expression from their tumor biopsies. Differences in overall survival rates were computed between patients with top 20% *SCD* RNA-Seq counts in their tumor samples and those with bottom 20% *SCD* expression, as Kaplan-Meier plots in all cancer patients and skin cancer patients (SKCM) from the TCGA dataset. (**e**) The boxplot shows average mRNA reads of *SCD* in 4,645 single cells from tumor samples of 19 melanoma patients (GSE72056). *SCD* expression was compared between malignant and nonmalignant cells.

Elevated lipogenic enzyme activities have been reported in colon, breast and prostate cancers^42–44^. Positive correlation of RNA and protein abundance of lipogenic enzymes was confirmed in breast cancer biopsies from Clinical Proteomic Tumor Analysis Consortium (CPTAC) (Supplemental Table 1)^45^. We analyzed the expression of five major DNFA enzymes *SCD*, *FASN* (Fig. 1a,b), *ACLY*, *ACSS2* (Supplementary Fig. 1a,b) and *ACACA* (Supplementary Fig. 2a) using RNA-Seq data from 30 diverse cancer types in The Cancer Genome Atlas (TCGA). We found that DNFA enzyme expression varies widely among cancers. Four DNFA enzymes – *SCD*, *FASN*, *ACLY* and *ACSS2* – exhibit the highest levels of mRNA expression in skin cutaneous melanoma (SKCM) compared to other tumor types, whereas expression of *ACACA* is less elevated in melanomas (Supplementary Fig. 2a). We observed relatively low expression of mRNAs encoding HMGCS1 and HMGCR, two rate-limiting enzymes in the *de novo* cholesterol synthesis (DNCS) pathway^46^ in melanomas. These results indicate that elevated DNFA expression is prevalent among tumors, significantly more so in melanomas than in most others.

To relate elevated DNFA expression with disease progression, we examined its prognostic value using Kaplan-Meier analysis. We divided patients into two groups based on the DNFA gene expression from their tumor biopsies and compared overall survival rates. We found that high expression of *SCD* (Fig. 1c and Supplementary Fig. 2d,g), *FASN* (Supplementary Figs 1c and 2e,h) and *ACACA* (Supplementary Fig. 2b,f) significantly correlates with poor prognosis in all cancers considered collectively. The only exceptions we observed were *ACLY* (Supplementary Fig. 1e) and *ACACA* specifically with a top 10% versus bottom 10% expression cutoff (Supplementary Fig. 2i). Elevated mRNA expression of *SCD* (Fig. 1d), *FASN*, *ACLY* (Supplementary Fig. 1d,f) and *ACACA* (Supplementary Fig. 2c) each correlates with poor prognosis in SKCM. We observed for *HMGCS1* but not *HMGCR* that higher expression significantly correlates with poor survival in all tumor types combined (Supplementary Fig. 3c,e), using 20% as cutoff, however, this was not observed in SKCM (Supplementary Fig. 3d,f).

Melanoma is the most aggressive skin cancer type. To control for potential tissue-specific upregulation, we first compared *SCD* expression in healthy tissues using RNA-Seq data from the Genotype-Tissue Expression (GTEx) database^47^. There, skin *SCD* expression is low (Supplementary Fig. 4a); yet among tumor tissues, skin *SCD* expression is elevated (Fig. 1a). This contrasts with, for example, the liver, which exhibits relatively high *SCD* expression among healthy (Supplementary Fig. 4a) and tumor tissues (Fig. 1a). We then compared *SCD, ACLY* and *HMGCS1* expression using normalized RNA-Seq data from the SKCM group in TCGA and the normal skin tissue group in GTEx. *SCD* and *ACLY* mRNA expression is markedly elevated in SKCM versus healthy skin tissues (Supplementary Fig. 4b,c), while *HMGCS1* is higher in the healthy tissues (Supplementary Fig. 4d). We performed principal component analysis (PCA) on RNA-Seq data of DNFA genes from skin tumors and healthy skin tissues (Supplementary Fig. 4e,f), and found that skin tumors are distinct from healthy skin samples. *SCD* expression strongly correlates with principal component 1 (PC1) towards skin tumors. Long-chain acyl-CoA synthetase (ACSL1) is a dual function enzyme in DNFA and fatty acid oxidation pathways with low abundance in tumors^4^. When its expression is combined with other DNFA genes in PCA, *ACSL1* expression strongly correlates with PC1 towards healthy tissues and results in better separation of skin tumors from healthy skin tissues (Supplementary Fig. 4e,f). In a separate PCA, DNCS expression, particularly *HMGCS1*, strongly correlates with PC1 (Supplementary Fig. 4g). Supplementary Fig. 4e–g suggests difference in lipogenic activities between skin tumors and healthy skin tissues.

Melanomas are transformed from benign, melanocytic nevi, with proliferation of melanocytes commonly triggered by *BRAF*^*V600E*^-activating mutation^48^. To confirm cancer-selective elevation in expression, we compared the DNFA gene expression between malignant melanoma biopsies and benign melanocytic nevus samples (Supplementary Fig. 5a–d). We observed significantly elevated DNFA gene expression in malignant melanomas. We further analyzed single cell RNA-Seq data from melanoma samples^49^^.^ High expression of *SCD*, *FASN*, and *ACACA* was confined to malignant cells, with low expression in healthy adjacent tissue (Fig. 1e and Supplementary Fig. 5e–h). Somatic mutations of *BRAF* and *NRAS* are associated with about 50% and 15% of melanomas, respectively^48^. *BRAF* and *NRAS* mutations, while being well-known risk factors and drivers of cancer onset, have limited prognostic significance for overall survival of melanoma patients^50^. There is no significant correlation between DNFA enzyme expression and common oncogenic driver mutations in SKCM (Supplementary Fig. 6a–l), suggesting that elevated DNFA expression in malignant melanomas is mechanistically unrelated to *BRAF* and *NRAS* mutation status.

### SREBP1 contributes to elevated DNFA gene expression in melanoma cells

**Figure 2:**
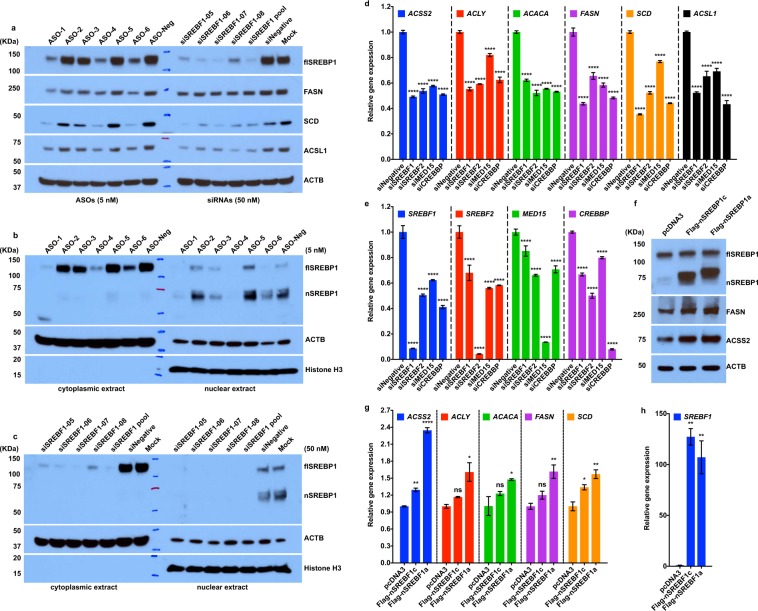
SREBP1 regulates the elevated DNFA gene expression in melanoma cells. (**a**) HT-144 cells were treated with ASOs, individual siRNA agents or pooled siRNAs (all individual agents combined) to deplete SREBP1 in 1% ITS medium. Total cell lysates were assayed with immunoblot by the indicated antibodies. HT-144 cells were transfected with (**b**) ASOs, (**c**) individual siRNA agents or pooled siRNAs in 1% ITS medium. Nuclear and cytoplasmic extracts were isolated for Western blot analysis of full length (fl) and nuclear (n) SREBP1 protein after treatment. (**d**,**e**) HT-144 cells were transfected with the pooled siRNAs (50 nM) in 1% ITS medium for three days to deplete *SREBF1*, *SREBF2*, *MED15* or *CREBBP*. RT-qPCR assay of mRNA shows relative expression of DNFA enzymes from siRNAs treatment groups to that of negative control siRNA treatment (siNegative). (**f**–**h**) HT-144 cells were transfected with plasmids carrying the transcriptionally active N-terminal portion of SREBP1a (nSREBP1a), N-terminal portion of SREBP1c (nSREBP1c) or empty vector (pcDNA3) for two days. (**f**) Total cell lysate was analyzed by Western blot assay using the indicated antibodies. (**g**,**h**) mRNAs were analyzed with RT-qPCR assay. The bar graphs show the relative expression of DNFA enzymes to that of pcDNA3 (control) transfection group. Data are expressed as mean ± SD and quantified from triplicates. One-way ANOVA tests were performed. ns, not significant; *P < 0.05; **P < 0.01; ***P < 0.001; ****P < 0.0001.

To test whether SREBP1 drives elevated DNFA enzyme expression in melanomas, we depleted the SREBP1 mRNA (*SREBF1*) with antisense oligonucleotides (ASOs) and siRNAs in HT-144 cells. *SREBF1* depletion with siRNA and ASO agents was accompanied by decreasing protein levels of SREBP1 and DNFA enzymes (Fig. 2a). The pooled siRNA and ASO agents effectively depleted both the cytoplasmic precursor and the mature nuclear SREBP1 (Fig. 2b,c). Among six tested ASOs targeting *SREBF1*, ASO-1 and ASO-4 are more potent than single or pooled siRNAs for *SREBF1*, as 5 nM of ASOs inhibited DNFA mRNA production as effectively as 50 nM of siRNAs (Supplementary Fig. 7b–f), even when siRNAs decreased the level of *SREBF1* mRNA more than ASOs (Supplementary Fig. 7a). ASOs may also engage in steric translation inhibition of *SREBF1* mRNA^51^, which could potentially contribute to their greater potency. We found that ASO-4 inhibits DNFA gene expression commensurately with dosage (Supplementary Fig. 7g).

To evaluate potential activators of DNFA expression, we depleted *SREBF1*, *SREBF2*, and co-activators *MED15* and *CREBBP*^28^ with siRNAs, and examined DNFA expression across three melanoma cell lines: HT-144 (Fig. 2d,e), A375 (Supplementary Fig. 7i,j) and MEL-JUSO (Supplementary Fig. 8a,b). We observed a similar range of mRNA reductions (50–70%) for most DNFA enzymes after *SREBF1* depletion (Fig. 2d, Supplementary Figs 7i and 8a). Depletion of *MED15* and *CREBBP* individually or together impacts DNFA gene expression, but to a lesser extent than *SREBF1* depletion (Supplementary Figs 7i and 8a). *SREBF2* depletion exerts potent effects on the expression of the DNCS genes *HMGCS1* and *HMGCR* in HT-144 and A375 cells (Supplementary Fig. 7h,k), in line with previous studies in hepatocytes^52^. *SREBF2* also affects DNFA enzyme expression, especially in concert with *SREBF1* depletion (Supplementary Fig. 8a–d). The role of SREBP2 in regulation of DNFA genes may be transitive via SREBP1^53^, since *SREBF1* expression decreases after *SREBF2* depletion (Fig. 2e); or it may be acting in a partially redundant manner^54^. Thus, we confirmed the transcription regulatory role of SREBP1 in promoting DNFA gene expression in multiple melanoma cell lines.

To understand the dynamics of DNFA gene expression, we performed a time-course study in A375 cells cultured under SREBP1-activating conditions (1% ITS medium)^55^. *FASN* and *SCD* displayed increased expression at consecutive time points (Supplementary Fig. 8c–f). *SREBF1* depletion by siRNA blocked DNFA gene activation on days 3 and 5, whereas DNFA enzyme protein (Fig. 2f) and mRNA levels (Fig. 2g,h) were elevated in response to overexpression of nSREBP1a, the abundant isoform in proliferating embryonic cells and in cancers, as well as nSREBP1c, which is the predominant isoform in adult liver and adipose tissues^56^. With a longer transactivation domain, nSREBP1a interacts more avidly with co-activators and thus exhibits stronger transcription activity than nSREBP1c^27,57^. Consistently, we observed higher expression of DNFA genes after overexpressing nSREBP1a than nSREBP1c. These results indicate that SREBP1 expression controls DNFA gene expression in melanoma cells.

### SREBP1 directly regulates DNFA pathway genes through RNAP II recruitment and productive elongation

**Figure 3:**
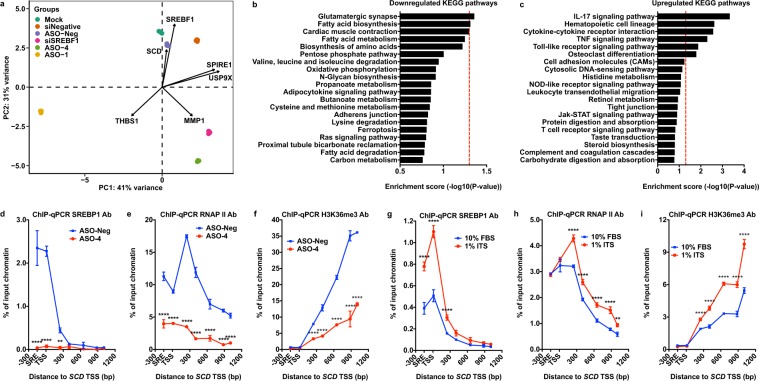
SREBP1 regulates DNFA pathway genes through RNAP II recruitment and productive elongation. (**a**) mRNAs of HT-144 cells were sequenced after ASOs (5 nM) or pooled siRNAs (50 nM) treatment in 1% ITS medium for three days. RNA-Seq data were analyzed with DESeq2 and principal component analysis (PCA). (**b**,**c**) The top 20 enriched signaling and metabolic KEGG pathways were discovered from differentially expressed genes (siSREBF1 group vs siNegative group) in RNA-Seq analysis using Generally Applicable Gene-set Enrichment (GAGE) method^106^. Red dash line marks P value = 0.05. (**d**–**f**) HT-144 cells were transfected with ASO-4 (5 nM) or the negative control ASO (5 nM), cultured in 1% ITS medium. Percentage of input DNA was compared between two treatments for the indicated antibodies at the 5′ promoter region of the *SCD* gene. (**g**–**i**) ChIP-qPCR analyses detected DNA pulldown using indicated antibodies at the *SCD* gene in HT-144 cells. ChIP-qPCR signals were compared between cells cultured in 10% FBS and 1% ITS medium conditions. Data were presented as mean ± SD and quantified from triplicates. Two-way ANOVA tests were performed. ns, not significant; *P < 0.05; **P < 0.01; ***P < 0.001; ****P < 0.0001.

To characterize the transcriptome changes after *SREBF1* depletion, we carried out RNA-Seq analysis after *SREBF1* depletion with pooled siRNAs and individual ASOs in HT-144 cells, followed by PCA on RNA-Seq data. To identify the possible off-target effect of the ASOs, we performed RNA-Seq analyses on two constructs that target different sequence regions of *SREBF1* mRNA: ASO-1 and ASO-4. The two principal components (PCs) in the PCA biplot represent over 70% of the overall gene expression changes (Fig. 3a and Supplementary Fig. 9a). *SREBF1* and *SCD* are among the top six contributors to data separation and they align with PC2 (Fig. 3a, Supplementary Fig. 9b). *SREBF1* siRNA, ASO-1 and ASO-4 produced similar vertical separations to all negative controls in PC2 (Fig. 3a), and they all reduced expression of *FASN* and *SCD* compared to negative controls (Supplementary Fig. 9e–g). These results indicate that the most profound effects after *SREBF1* depletion by both ASOs and siRNA are on DNFA genes. ASO-1 has significant lateral separation from ASO-4 and *SREBF1* siRNA on PC1 (Fig. 3a). The changes in *SPIRE1* and *USP9X* expression are the major contributors to PC1 (Supplementary Fig. 9c) and are only affected by ASO-1 (Supplementary Fig. 9h,i). This result indicates that ASO-1 has specific off-target effects on *SPIRE1* and *USP9X*. Multiple negative controls are grouped together, and ASO-4 is close to *SREBF1* siRNA on the PCA biplot, consistent with the results from hierarchical clustering analysis of the same RNA-Seq dataset (Supplementary Fig. 9d). Hence, ASO-4 has similar specificity as pooled *SREBF1* siRNAs for *SREBF1* depletion.

To explore the overall biological pathways affected by *SREBF1* depletion, we examined differentially expressed genes (DEGs) in the RNA-Seq dataset (siSREBF1 group vs siNegative group) (Supplementary Fig. 9j). Using gene-set enrichment analyses (GSEA) with Kyoto Encyclopedia of Genes and Genomes (KEGG) pathways and gene ontology (GO) terms on DEGs, we determined that the downregulated genes after *SREBF1* depletion are primarily involved in fatty acid biosynthesis and lipid metabolism (Fig. 3b, and Supplementary Fig. 9k), as expected. Cellular inflammatory response pathways are significantly enriched in upregulated genes after *SREBF1* depletion (Fig. 3c, and Supplementary Fig. 9l), including Toll like receptor and tumor necrosis factor (TNF) signaling pathways that mediate cytotoxicity^58^.

To assess the direct regulatory roles of SREBP1 on target genes in cancers, we analyzed public ChIP-Seq data for SREBP1 from lung cancer, breast cancer and chronic myeloid leukemia (CML) cell lines. ChIP-Seq peaks primarily localize at the proximal promoter regions around TSS (Supplementary Fig. 10a,b). *De novo* motif sequences identified from SREBP1 ChIP-Seq peaks match the known SREBP1 binding motif (Supplementary Fig. 10c). High DNFA gene expression was observed in lung and breast cancers (Fig. 1a). We identified genes that are present in SREBP1 ChIP-Seq data from both lung cancer line A549 and breast cancer line MCF7 (Supplementary Fig. 10d), and that are also DEGs in our RNA-Seq data. We reasoned that the “overlapping” genes common to all three data sets are likely regulated directly by SREBP1. We therefore performed functional network analysis on this subset. This analysis revealed genes in many pathways including the PI3K/AKT pathway and the RNAP II elongation complex, and the expected DNFA pathways (Supplementary Fig. 10e). We then used our RNA-Seq data to divide the overlapping genes into upregulated and downregulated sets, and performed GSEA on each. The downregulated genes participate primarily in lipid metabolism pathways (Supplementary Fig. 10f), which confirms SREBP1 as a direct activator of DNFA genes. Inflammatory response pathways are significantly upregulated in DEGs from RNA-Seq analysis (Fig. 3c), but seem not to be direct targets of SREBP1 (Supplementary Fig. 10g). We suspect that downregulation of DNFA pathways may change the homeostasis of cellular fatty acids and exert further impact on inflammatory response pathways as well as cell death^59^.

To elucidate the molecular mechanism of SREBP1-governed DNFA gene activation in melanoma cells, we used a chromatin immunoprecipitation (ChIP)-qPCR assay to detect occupancy of SREBP1, RNA polymerase II (RNAP II), and H3K36me3 – a histone marker associated with transcription elongation^60^ on DNFA genes. SREBP1 depletion by ASO-4 diminished SREBP1, RNAP II and H3K36me3 signals at the *SCD* promoter (Fig. 3d–f). We observed similar (albeit lower-magnitude) results for *FASN* (Supplementary Fig. 11a–c). These ChIP-qPCR results together with the RNA-Seq data (Supplementary Fig. 9e–g) suggest that removal of SREBP1 at DNFA promoters inhibits transcription activity and mRNA production. We performed ChIP-qPCR analyses in SREBP1-activating (1% ITS medium, no lipids) and SREBP1–repressing (10% FBS medium, with lipids) conditions. We found that 1% ITS medium dramatically increases SREBP1 occupancy at the transcription start sites (TSS) of *SCD* (Fig. 3g) and *FASN* (Supplementary Fig. 11f) in HT-144 cells. The strong RNAP II binding peaks at TSS of *SCD* and *FASN* in both 10% FBS and 1% ITS culture conditions (Fig. 3h and Supplementary Fig. 11g) indicate promoter-proximal pausing of RNAP II^61^. Furthermore, culturing cells in 1% ITS medium increased the occupancy of actively elongating RNAP II (RNAP II S2P) (Supplementary Fig. 11d,i), but not poised RNAP II (RNAP II S5P)^62^ (Supplementary Fig. 11e,j), at the TSS as well as the gene bodies of both *SCD* and *FASN*. Accordingly, the histone marker H3K36me3 was elevated along both gene bodies in cells cultured with 1% ITS (Fig. 3i and Supplementary Fig. 11h). These results suggest that SREBP1 binding near the TSS involves both RNAP II recruitment and stimulation of productive elongation on DNFA genes.

### Essential role of DNFA in proliferation and survival of untreated and drug-resistant melanoma cells

**Figure 4:**
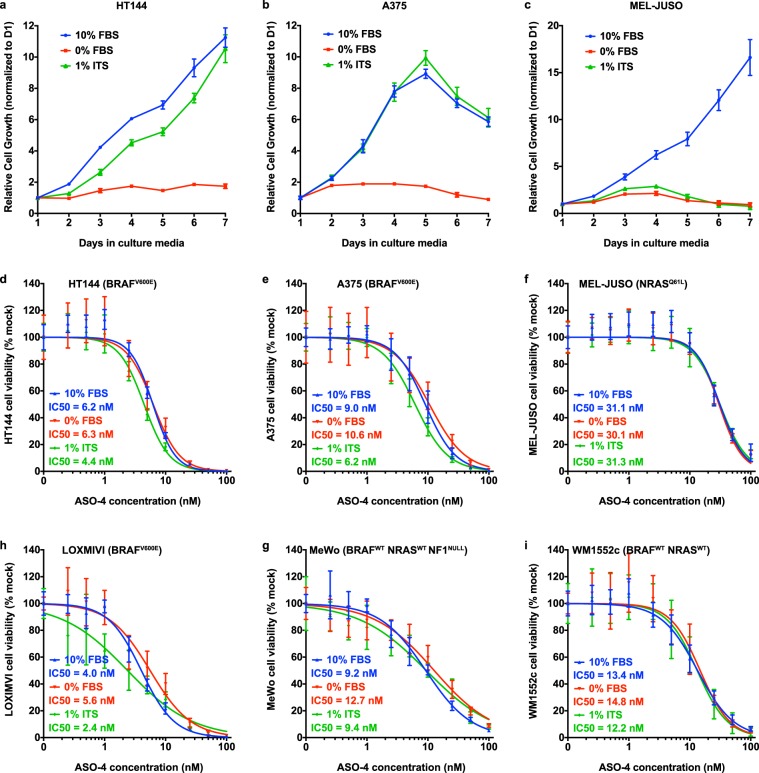
Reliance on DNFA for melanoma cell survival. (**a**–**c**) Cell growth of three melanoma cell lines was measured with cell-titer glo assay in 10% FBS, 0% FBS or 1% ITS medium in a time-course experiment. Cell proliferation was measured by cell-titer glo assay every day for seven days in three medium conditions. (**d**–**i**) Melanoma cell lines were transfected with various doses of ASO-4. Cell viability was measured by cell-titer glo assay six days after ASO transfection in three medium conditions. ASO-4 treatment diminished viability in quiescent cells, which was observed on cells cultured in 0% FBS medium. (**a–c**) LOXIMVI is the most sensitive line to ASO-4 treatment in all three culture conditions, while MEL-JUSO is the least sensitive line.

To evaluate the roles DNFA in cell proliferation and survival, we cultured melanoma cells in the lipid-free 1% ITS medium, using insulin as a growth factor to stimulate proliferation. The metastatic melanoma-derived cell lines HT-144 and A375 can proliferate in both 10% FBS and 1% ITS media but remain quiescent in 0% FBS medium (Fig. 4a,b). By contrast, the primary melanoma-derived cell line, MEL-JUSO, remains quiescent in both lipid-free (1% ITS and 0% FBS) media (Fig. 4c). We depleted *SREBF1* with ASO-4 in several melanoma cell lines cultured under the three medium conditions, to obtain dose-response curves and IC50 values. We found that ASO-4 decreased viability of both proliferative and quiescent cells in all conditions (Fig. 4d–i). We reason that, although cancer cells may employ lipid uptake^63^, DNFA is required for cell survival regardless of external lipid availability under conditions similar to those we tested. We observed diminished viability at higher ASO concentrations (greater than 5 nM), which correlates with more efficient depletion of SREBP1 and more marked decrease of DNFA gene expression (Supplementary Fig. 7g). Comparing the two growth factor-containing culture conditions, we find that ASO-4 is a more potent inhibitor of cell growth/survival in 1% ITS medium than in 10% FBS medium with HT-144, A375 and LOXIMVI cells. In contrast, WM1552C, MeWo and MEL-JUSO cells are relatively insensitive to ASO-4 treatment. The implication is that, although DNFA seems to be vital for cell survival, medium lipid availability may mitigate the impact of SREBP1 inhibition in cell lines that are sensitive to ASO-4.

**Figure 5:**
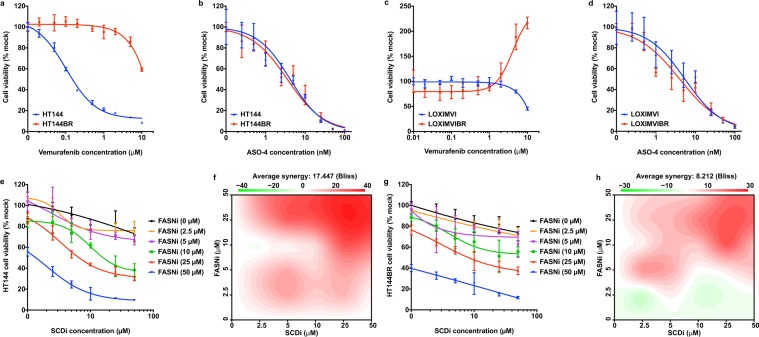
DNFA supports survival in drug-resistant melanoma cells. (**a**,**b**) HT-144BR is a BRAF inhibitor resistant HT-144 cell line, which was derived after prolonged treatment with vemurafenib (2 μM) in 10% FBS medium for three months. Viability of HT-144 and HT-144BR cells responding to vemurafenib or ASO4 treatment in 10% FBS medium was assessed by cell titer-glo assay. (**c**,**d**) LOXIMVIBR is the BRAF inhibitor resistant LOXIMVI cell line, which was derived after prolonged treatment with vemurafenib (2 μM) in 10% FBS medium for three months. Viability of LOXIMVI and LOXIMVIBR cells responding to vemurafenib or ASO-4 treatment was assessed by cell titer-glo assay in 10% FBS medium. (**e**–**h**) HT-144 and HT-144BR cells were co-treated with FASN inhibitor (FASNi) GSK 2194069 and SCD inhibitor (SCDi) MF-438 in 10% FBS medium or 10% FBS medium with vemurafenib (2 μM). (**e**,**g**) Cell viability was measured by cell titer-glo assay six days after treatment. (**f**,**h**) The combination responses for two DNFA enzyme inhibitors were evaluated with the Bliss independence model. Positive Bliss scores indicate synergy between two drugs.

BRAF inhibitors (BRAFi, e.g. vemurafenib) improve survival for patients with metastatic melanomas harboring the *BRAF*^*V600E*^ mutation^64^. However, the rapid emergence of treatment-surviving tumors limits clinical benefits^65,66^. We derived two BRAFi-resistant cell lines with prolonged vemurafenib treatment: HT-144BR from a vemurafenib-sensitive cell line HT-144, and LOXIMVIBR from a vemurafenib-insensitive cell line LOXIMVI (Fig. 5a,c). Both HT-144BR and LOXIMVIBR cell lines continuously proliferate in the presence of vemurafenib (2 μM). Using cell viability assay, we found that HT-144 and HT-144BR displayed similar sensitivities to ASO-4 treatment, as did LOXIMVI and LOXIMVIBR (Fig. 5b,d). We reason that these cell lines all depend on SREBP1 for survival regardless of whether they are sensitive or resistant to vemurafenib.

To confirm that DNFA enzyme inhibition is the crucial driver for reduced cell viability, we then used small molecule inhibitors of FASN and SCD enzymes, which reportedly decreased tumor growth in preclinical studies^67–69^. In 10% FBS (lipid-containing) medium, FASN and SCD inhibitors decreased viability in both HT-144 and HT-144BR cells (Fig. 5e,g), although the effect was less potent than *SREBF1* depletion by ASO-4. However, we observed much stronger effects on cell survival when combining the two inhibitors, confirmed by Bliss independence analysis^70^ as robust positive synergy (Fig. 5f,h). DNFA thus appears vital to melanoma cell survival even in vemurafenib-resistant cells.

### Continued and elevated DNFA gene expression in melanoma cells after BRAF inhibitor treatment

**Figure 6:**
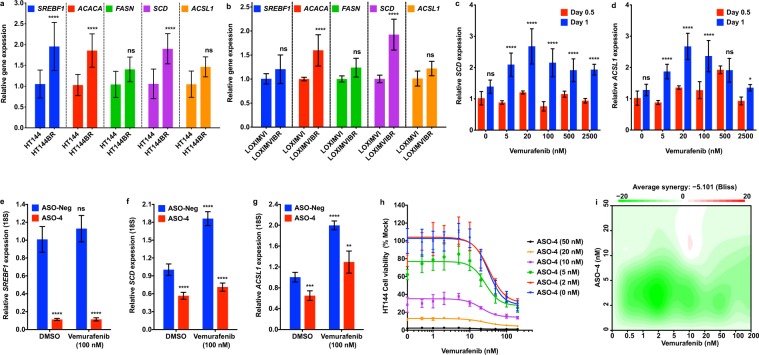
Elevated DNFA expression accompanies drug resistance in BRAFi treated melanoma cells. (**a**) RT-qPCR assay compared the DNFA gene expression between HT-144 cells in 10% FBS medium and HT-144BR cells in 10% FBS medium with vemurafenib (2 μM). (**b**) RT-qPCR assay compared the DNFA gene expression between LOXIMVI cells in 10% FBS medium and LOXIMVIBR cells in 10% FBS medium with vemurafenib (2 μM). (**c**,**d**) HT-144 cells were treated with different dosages of vemurafenib for 0.5 or 1 day in 1% ITS medium. *SCD* expression was assayed by RT-qPCR analysis. Expression of *SCD* from all treatment groups was normalized to expression under DMSO treatment at day 0.5 (normalized as 1). Relative gene expression was compared between 0.5-day and 1-day treatment groups. Data were presented as mean ± SD and quantified from triplicates. Two-way ANOVA tests were performed. *P < 0.05; **P < 0.01; ***P < 0.001; ****P < 0.0001. (**e**–**g**) HT-144 cells were transfected with ASO-4 (5 nM) and then treated with vemurafenib (100 nM) in 1% ITS medium for one day. RT-qPCR assay analyzed DNFA gene expression from treatment groups relative to expression under ASO-Neg and DMSO treatment at day 1 (normalized as 1). Data are presented as mean ± SD and quantified from triplicates. One-way ANOVA tests were performed. ns, not significant; *P < 0.05; **P < 0.01; ***P < 0.001; ****P < 0.0001. (**h**) HT-144 cells were transfected with ASO-4, and then treated in combination with vemurafenib. Cell viability was measured by cell titer-glo assay three days after combined treatment in 1% ITS medium. (**i**) The drug interaction in (**h**) was evaluated with the Bliss independence model.

To explore the role of DNFA in BRAFi resistance, we compared DNFA gene expression in untreated and vemurafenib-resistant cell lines. We observed modestly higher DNFA gene expression in the vemurafenib-resistant HT-144BR and LOXIMVIBR cells as compared with the untreated HT-144 and LOXIMVI cells (Fig. 6a,b). We then investigated the short-term impact of vemurafenib on DNFA gene expression in HT-144 cells treated with various doses of vemurafenib. After one-day treatment, vemurafenib exerted low-dose stimulation and high-dose inhibition of DNFA gene expression, exhibiting hormesis (i.e. bell-shaped dose-response curves)^71^ (Fig. 6c,d, Supplementary Fig. 12a–e). We observed dose–dependent induction of *PPARGC1A* expression with vemurafenib treatment (Supplementary Fig. 12f) as expected, given that the BRAF/MEK pathway directly suppresses *PPARGC1A* expression and fatty acid oxidation in melanomas^72,73^. DNFA inhibition in response to high-dose vemurafenib treatment may correlate with onset of cell death, whereas DNFA stimulation in response to low-dose vemurafenib treatment suggests rapid cellular resistance.

To rule out vemurafenib-specific (perhaps off-target) effects, we evaluated dabrafenib, another BRAFi employed in clinical practice^74^. We monitored DNFA gene expression in HT-144 cells after one day treatment of dabrafenib. Cell death was less evident after high-dose dabrafenib treatment, perhaps due to lower off-target toxicity^75^. We found that expression of DNFA genes decreased after low-dose dabrafenib treatment but increased at effective high-dose treatment (Supplementary Fig. 13a–g). Dabrafenib at 2.5 µM concentration significantly stimulated gene expression of *ACLY*, *SCD*, *ACSL1* and *PPARGC1a* (Supplementary Fig. 13b,e,f and h).

The effectiveness of BRAF inhibition is widely attributed to downstream inhibition of MEK, and then ERK. Resistance due to reactivation of MEK/ERK bypasses that effect. In A375 cells (Supplementary Fig. 14a–h), a cell line with reported MEK/ERK reactivation associated with vemurafenib treatment^76^, vemurafenib exerts little induction of DNFA gene expression nor dose–dependent induction of *PPARGC1A*. An adjuvant ERK inhibitor (ERKi, SCH772984) has been employed clinically to overcome vemurafenib resistance^77^, by directly contributing to overall BRAF/MEK/ERK pathway inhibition^78^. When combining 0.5 μM vemurafenib and 0.5 μM SCH772984 for treatment, mimicking clinical practice, we observed induction of DNFA in A375 cells (Supplementary Fig. 14i). We saw evidence of a dose-response relationship between ERKi and induction of DNFA gene expression, where treatment of SCH772984 at 1 μM achieved stronger induction of DNFA gene expression (Supplementary Fig. 14i). Our overall interpretation is that BRAF/MEK/ERK pathway inhibition upregulates DNFA gene expression, possibly through AKT activation^79^, which then contributes to vemurafenib tolerance in melanoma cells.

To further characterize the role of SREBP1 and DNFA upregulation in response to BRAF inhibition, we combined ASO-4 and vemurafenib treatment in HT-144 cells. We observed that *SREBF1* depletion abolished the *SCD* and *ASCL1* induction accompanying vemurafenib treatment by RT-qPCR assays (Fig. 6e–g). Finally, we investigated cell viability after co-treatment with ASO-4 and vemurafenib. ASO-4, alone or in combination with vemurafenib, effectively killed HT-144 (Fig. 6h) and A375 cells (Supplementary Fig. 14j). However, there is a mild antagonistic effect between ASO-4 and vemurafenib at low doses according to Bliss analysis (Fig. 6i, Supplementary Fig. 14k). We regard this as corollary to DNFA stimulation by vemurafenib (Fig. 6e–g, Supplementary Fig. 14a–g), while noting that, consistent with our findings above for A375, vemurafenib treatment alone yielded little induction of DNFA gene expression in A375 compared to HT-144, and the antagonistic effect was correspondingly lower. In both cell lines, high-dose ASO-4 exerts dominant cell killing effect over vemurafenib in combination treatment.

## Discussion

Cancers frequently exhibit reprogrammed metabolic traits such as elevated DNFA^3^ that act to sustain active proliferation and cell survival under adverse conditions, and support the process of tumorigenesis and metastasis, as well as resistance to targeted therapies. The diverse genetic paths cancers take to achieve such traits have frustrated efforts to exploit them clinically^80–82^.

Most DNFA enzymes are primarily regulated at the transcriptional level in a coordinated manner^6^, thus the abundance of their mRNAs can be employed as a simple surrogate for DNFA activities. Our results show that elevated DNFA gene expression may serve as a prognostic marker for some cancer types, even though they are not considered onco-drivers. Moreover, increased DNFA pathway expression appears to be intrinsic to malignant cancer cell types independently of onco-drivers, including constitutively active *BRAF* mutants. This may be consistent with the notion of increased DNFA as an “oncosustenance” pathway, with well-definable mechanistic contributions to cancer survival and proliferation. Once the oncosustenance pathway becomes active, it may not always matter which onco-drivers acted prior to cancer onset. Indeed, it has already been suggested that malignant cancer cells may have ongoing reliance upon oncogene-induced signaling pathways, but not upon initial onco-drivers^83^. It is also possible that SREBP1 transcription auto-regulation^28^ might “lock in” elevated DNFA expression in malignant cells. The scope of therapeutic investigations, which frequently focus on mutated oncogenes, could be broadened to include oncosustenance mechanisms.

The prevalence of elevated DNFA expression in certain tumor types such as melanoma suggests the presence of survival pressure for increased lipogenesis, which may therefore represent a therapeutic linchpin. However, high doses of individual pharmacological inhibitors of SCD and FASN enzymes were reportedly required to impact cell viability in melanomas and other cancers^67,69^. This phenomenon may be attributable to compensatory upregulation of SREBP1 and corresponding elevation of DNFA pathway expression, triggered by individual DNFA enzyme inhibition^84^. However, the combined effect of multiple DNFA enzyme inhibitors is synergistic and potent, at least *in vitro* (Fig. 5e–h). We demonstrated in melanomas that inhibition of *SREBF1* by 5 nM ASO-4 treatment results in 50% reduction in the expression of multiple DNFA genes. The potent cell death effect after ASO-4 treatment is likely due to the strong synergistic effects of 50% reduction across the entire DNFA enzymatic cascade that is coordinately regulated by SREBP1. Therefore, inhibition of multiple DNFA enzymes could be of therapeutic benefit due to a potent cumulative effect on lipogenesis, as observed with SREBP1 mRNA-inhibiting ASOs and DNFA enzyme-targeting cocktails.

Previous studies have shown that SREBP1 binding and RNAP II recruitment to DNFA gene promoters represents the primary mechanism for transcription activation^85,86^. In accord with this, we observed that RNAP II accumulated at the proximal promoter regions of *SCD* and *FASN*. Under lipid-depleted (SREBP1-activating) cell culture conditions, we observed two indications of productive transcription elongation: increased RNAP II with serine 2 phosphorylation at its C-terminal domain (RNAPII-S2p)^62^ and the histone marker for transcription elongation (H3K36me3)^87^ along the gene bodies of *FASN* and *SCD*. Based on these findings we suggest a two-step working model for regulation of RNAP II machinery: RNAP II recruitment to proximal promoters at lipogenic genes, followed by RNAP II release for productive elongation. This model appears to explain elevated and synchronous DNFA gene expression in melanomas, and perhaps other cell types. It is presently unclear exactly how RNAP II is released for productive elongation.

BRAF-mutated melanomas are frequently treated with vemurafenib, a targeted therapy to inhibit the oncogenic BRAF pathway. Because vemurafenib sometimes fails to inhibit MEK/ERK (key targets downstream of BRAF)^88^, current clinical regimens combine inhibitors of both BRAF and MEK and/or ERK for treating metastatic melanomas^89,90^. However, even when combined inhibition is achieved, resistance arises via genetic alterations that upregulate the PI3K/AKT pathway^91^. Our determination that elevated DNFA is mechanistically important for resistance to targeted therapies dovetails with a similar recent finding by Talebi *et al*.^92^. They reported that SREBP1 processing is downregulated in vemurafenib-responsive cells, while BRAFi-resistant melanoma cells maintain SREBP1 processing to protect them from ROS-induced cell death. We find that (1) BRAFi treatment is associated with DNFA stimulation in a dose-response relationship and (2) elevated DNFA gene expression is a vital survival trait of melanoma cells both before and after they achieve resistance to combined BRAF/MEK inhibition. A speculative explanation is that, compared to cells vulnerable to BRAFi treatment, resistant cells achieve a state that relies more on DNFA activities and less on BRAF activities. We suspect that increased PI3K/AKT signaling activates SREBP1 and DNFA for survival in BRAFi-resistant melanoma cells^79^. There is ongoing investigation to demonstrate mechanisms of cell state-related drug resistance.

In summary, our work demonstrates that melanomas engage the DNFA pathway for cell survival – more so during drug resistance – and employs SREBP1 to promote transcription activation and elongation of DNFA enzyme genes. Although immunotherapy has emerged as a highly promising treatment for a subset of melanoma patients^93^, it may be hampered by significant complications^94^. SREBP1 and/or DNFA enzyme inhibition may represent potential therapeutic alternatives worthy of further exploration for the treatment of melanoma and possibly other cancer types characterized by elevated DNFA.

## Methods

### Cell culture and reagents

The human melanoma cell lines HT-144, MEL-JUSO, LOXIMVI, WM1552C and MeWo were kindly provided by C. Benes at MGH. A375 cell line was purchased from American Type Culture Collection (ATCC). All melanoma cell lines were regularly maintained and passaged in a humidified incubator at 37 °C with 5% CO_2_, in RPMI-1640 medium (21870092, Thermo Fisher Scientific) supplemented with 10% fetal bovine serum^95^ (Gibco), plus 2 mM L-glutamine (Gibco) and 50 U/ml penicillin-streptomycin (Gibco). Two types of lipid-free medium were used for assays: 0% FBS medium contained the RPMI-1640 medium supplemented with 2 mM L-glutamine (Gibco) and 50 U/ml penicillin-streptomycin (Gibco); 1% ITS medium contained the RPMI-1640 medium supplemented with 1 x Insulin-Transferrin-Selenium (ITS-G, Thermo Fisher Scientific), 2 mM L-glutamine (Gibco) and 50 U/ml penicillin-streptomycin (Gibco).

The BRAF inhibitor vemurafenib (S1267) and ERK inhibitor SCH772984 (S7101) were purchased from Selleck Chemicals. Dabrafenib was kindly provided by R. Corcoran at MGH. SCD inhibitor MF-438 (569406, Sigma) and FASN inhibitor GSK 2194069 (5303, Tocris) were dissolved in dimethyl sulfoxide (DMSO) to yield 50 mM stock solutions for *in vitro* studies. To generate vemurafenib-resistant cells, parental cells were exposed to increasing concentrations of vemurafenib (from 1 μM to 2 μM) for three months. The resistance was confirmed by measuring cell viability under vemurafenib treatment.

### TCGA data analysis

We analyzed 10,210 TCGA samples from 30 cancer types, for which RNA-Seq data were publically available. Briefly, gene-level RNA-Seq expression data (normalized RSEM (RNA-seq by expectation-maximization) value) were obtained from cBioportal (http://www.cbioportal.org). For Kaplan-Meier plots, RNA-Seq expression data and patient survival data from TCGA all cancers data set (10,210 samples) or TCGA skin cutaneous melanoma (SKCM) data set (476 samples) were obtained from UCSC Xena (https://xenabrowser.net). To compare gene expression in normal skin tissues and skin tumors (Fig. S2b, c), we used the normalized RNA-Seq data of TCGA and GTEx (by TOIL and DESeq2 methods^96^) from UCSC Xena. To compare gene expression with oncogenic mutations in TCGA skin tumor study groups (Supplementary Fig. 4), RNA-Seq and oncogene mutation data were obtained and analyzed using CGDS R package^97^.

### Plasmids, siRNAs and ASOs

pcDNA3-Flag-nSREBP1a (plasmid #26801) and pcDNA3-Flag-nSREBP1c (plasmid #26802) were purchased from Addgene^57^. HT-144 cells were transfected with plasmids with Lipofectamine 2000 reagent (Thermo Fisher Scientific). The human-specific siRNAs targeting *SREBF1* (6720), *SREBF2* (6721), *MED15* (51586) and *CREBBP* (1387) were pre-designed ON-TARGETplus SMARTpool siRNA reagents from Dharmacon. Each ON-TARGETplus SMARTpool siRNA was a mixture of four siRNA duplexes. Sequences of individual siRNAs in each SMARTpool reagent were as follows:

siSREBF1-05 (J-006891-05): 5′-GCGCACUGCUGUCCACAAA-3′

siSREBF1-06 (J-006891-06): 5′-GAAUAAAUCUGCUGUCUUG-3′

siSREBF1-07 (J-006891-07): 5′-CGGAGAAGCUGCCUAUCAA-3′

siSREBF1-08 (J-006891-08): 5′-GCAACACAGCAACCAGAAA-3′

siSREBF2-05 (J-009549-05): 5′-GGACAGCGCUCUGGCAAAA-3′

siSREBF2-06 (J-009549-06): 5′-GCACACUGGUUGAGAUCCA-3′

siSREBF2-07 (J-009549-07): 5′-GCAGUGUCCUGUCAUUCGA-3′

siSREBF2-08 (J-009549-08): 5′-GAAAGGCGGACAACCCAUA-3′

siMed15-09 (J-017015-09): 5′-CCAAGACCCGGGACGAAUA-3′

siMed15-10 (J-017015-10): 5′-GGGUGUUGUUAGAGCGUCU-3′

siMed15-11 (J-017015-11): 5′-GGUCAGUCAAAUCGAGGAU-3′

siMed15-12 (J-017015-12): 5′-CCGGACAAGCACUCGGUCA-3′

siCREBBP-06 (J- 003477-06): 5′-GCACAGCCGUUUACCAUGA-3′

siCREBBP-07 (J-003477-07): 5′-UCACCAACGUGCCAAAUAU-3′

siCREBBP-08 (J-003477-08): 5′-GGGAUGAAGUCACGGUUUG-3′

siCREBBP-09 (J-003477-09): 5′-AAUAGUAACUCUGGCCAUA-3′

The negative control siRNAs were the ON-TARGETplus Non-Targeting Pool reagents (D-001810-10, Dharmacon). Single-stranded antisense oligonucleotides (ASOs) for SREBF1 were designed and purchased as the locked nucleic acid (LNA) gapmers with phosphorothioate bonds from Exiqon. Sequences of individual ASOs were as follows:

ASO-Neg: 5′-+C* + G* + A*A*T*A*G*T*T*A*G*T*A* + G* + C* + G-3′

ASO-1: 5′- + G* + C* + G*C*A*A*G*A*C*A*G*C* + A* + G* + A* + T-3′

ASO-2: 5′- + T* + A* + A*G*G*G*G*A*G*T*T*A* + A* + C* + G* + G-3′

ASO-3: 5′- + T* + A*A*G*G*T*T*T*A*G*A*G* + G* + G* + T* + G-3′

ASO-4: 5′- + C* + T* + T* + A*G*G*G*T*C*A*A*G*A*T* + C* + G-3′

ASO-5: 5′- + C* + A* + G*C*A*G*A*T*T*T*A*T* + T* + C* + A* + G-3′

ASO-6: 5′-+A* + C* + C* + G*T*A*G*A*C*A*A*A* + G* + A* + G* + A-3′

+ indicates a 2′-O, 4′-C-methylene-linked bicyclic ribonucleoside (LNA). * indicates a phosphorothioate internucleotide linkage.

siRNAs were suspended in RNase-free 1x siRNA Buffer (Dharmacon) to yield 20 μM stock solutions. ASOs were suspended in sterile ddH_2_O for stock solutions. Melanoma cell lines were transfected with siRNAs or ASOs using Lipofectamine RNAiMAX transfection reagent (Thermo Fisher Scientific) and the reverse transfection protocol suggested by the manufacture. Cells were subjected to immunoblotting or RT-qPCR analyses three days after transfection.

### Reverse transcription quantitative PCR (RT-qPCR) and RNA-Seq assays

RNAs were isolated from cultured cells using the RNeasy Mini Kit (Qiagen). RNAs were treated with RNase-free DNase (Qiagen). RNA concentrations were quantified with Qubit RNA BR Assay Kit (Thermo Fisher Scientific). One μg RNAs were used for cDNA synthesis with RNA to cDNA EcoDry Premix (TaKaRa) containing both random hexamer and oligo(dT)_18_ primers (Double Primed). qPCR was carried out in triplicates on a LightCycler 480 (Roche) using LightCycler 480 SYBR Green I Master (Roche). qPCR primers were designed by MGH primer bank (https://pga.mgh.harvard.edu/primerbank/) and the primer sequences are listed in Table 1. Relative gene expression was calculated with the 2^−ΔΔCt^ method^98^, and normalized to the 18S housekeeping gene. The mean of negative control samples was set to 1.

**Table 1:** The primers used for RT-qPCR assays.

ACACA forward	5′-ATGTCTGGCTTGCACCTAGTA-3′
ACACA reverse	5′-CCCCAAAGCGAGTAACAAATTCT-3′
ACLY forward	5′-TCGGCCAAGGCAATTTCAGAG-3′
ACLY reverse	5′-CGAGCATACTTGAACCGATTCT-3′
ACSS2 forward	5′-AAAGGAGCAACTACCAACATCTG-3′
ACSS2 reverse	5′-GCTGAACTGACACACTTGGAC-3′
FASN forward	5′-AAGGACCTGTCTAGGTTTGATGC-3′
FASN reverse	5′-TGGCTTCATAGGTGACTTCCA-3′
SCD forward	5′-TCTAGCTCCTATACCACCACCA-3′
SCD reverse	5′-TCGTCTCCAACTTATCTCCTCC-3′
ACSL1 forward	5′-CCATGAGCTGTTCCGGTATTT-3′
ACSL1 reverse	5′-CCGAAGCCCATAAGCGTGTT-3′
SREBF1 forward	5′-ACAGTGACTTCCCTGGCCTAT-3′
SREBF1 reverse	5′-GCATGGACGGGTACATCTTCAA-3′
SREBF2 forward	5′-AACGGTCATTCACCCAGGTC-3′
SREBF2 reverse	5′-GGCTGAAGAATAGGAGTTGCC-3′
HMGCS1 forward	5′-GATGTGGGAATTGTTGCCCTT-3′
HMGCS1 reverse	5′-ATTGTCTCTGTTCCAACTTCCAG-3′
HMGCR forward	5′-TGATTGACCTTTCCAGAGCAAG-3′
HMGCR reverse	5′-CTAAAATTGCCATTCCACGAGC-3′
LDLR forward	5′-ACCAACGAATGCTTGGACAAC-3′
LDLR reverse	5′-ACAGGCACTCGTAGCCGAT-3′
Med15 forward	5′-ATGGACGTTTCCGGGCAAG-3′
Med15 reverse	5′-GCATCCTCGATTTGACTGACCA-3′
CREBBP forward	5′-CAACCCCAAAAGAGCCAAACT-3′
CREBBP reverse	5′-CCTCGTAGAAGCTCCGACAGT-3′
18S forward	5′-GTAACCCGTTGAACCCCATT-3′
18S reverse	5′-CCATCCAATCGGTAGTAGCG-3′

For RNA-Seq, harvested RNA samples were treated with RNase-free DNase (Qiagen). RNA quality was verified with RNA ScreenTape (Agilent) on an Agilent 2200 TapeStation (Agilent). mRNAs were isolated from total RNAs for library preparation using NEBNext Poly(A) mRNA Magnetic Isolation Module (NEB). RNA-Seq libraries were generated using NEBNext Ultra Directional RNA Library Prep Kit for Illumina (NEB) and quantified using KAPA Library Quantification Kit (KAPABiosystem). Adaptor indexed strand-specific RNA-Seq libraries were pooled and sequenced using the pair-end 75-bp per read setting for NextSeq500 High Output v2 Kit (150 cycles) on the NextSeq 500 sequencer (Illumina).

### Immunoblotting assay

Total cell lysate was prepared with RIPA buffer with protease inhibitors (Protease Inhibitor Cocktail Tablets, Roche). Nuclear and cytoplasmic protein fractions were prepared with NE-PER Nuclear and Cytoplasmic Extraction Reagents (Thermo Fisher Scientific). Protein samples were separated on the SDS-PAGE gels using 4-15% Mini-PROTEAN TGX Precast Gels (Bio-Rad) and then transferred to polyvinyl difluoride (PVDF) membranes (Immobilon-P, Millipore) for immunoblotting analysis. The following primary antibodies were used: mouse anti-SREBP1 (IgG-2A4, BD Biosciences), rabbit anti-FASN (C20G5, Cell Signaling), rabbit anti-SCD (23393-1-AP, Proteintech), rabbit anti-ACSL1 (D2H5, Cell Signaling), rabbit anti-ACSS2 (D19C6, Cell Signaling), rabbit anti-histone H3 (9715, Cell Signaling) and rabbit anti-actin (13E5, Cell Signaling). After being incubated with primary antibodies overnight in PBST solution with 5% non-fat dry milk, membranes were probed with HRP-conjugated affinity-purified donkey anti-mouse or anti-rabbit IgG (GE Healthcare) and visualized using the Immobilon Western Chemiluminescent HRP Substrate (Millipore).

### RNA-Seq and ChIP-Seq analyses

For RNA-Seq, Illumina sequencing reads (FASTQ files) were checked with FASTQC for quality control and then aligned to the human genome (GRCh38.86). Genome index generation and sequence alignment were performed using STAR software^99^, followed by sorting and indexing of BAM files with SAMtools. Raw counts of reads mapped to genes were calculated using HT-Seq^100^. Differential expression analysis was performed using DESeq2 R package^101^. KEGG pathway analysis of differentially expressed gene lists and principal component analysis (PCA) were performed using functions within DESeq2. The complete RNA-Seq data set has been submitted to the National Center for Biotechnology Information (NCBI) in the Gene Expression Omnibus (GEO) database (series entry GSE122707).

SREBP1 ChIP-Seq in multiple cancer cell lines were previously published^102^. Bam files of SREBP1 and IgG control ChIP-Seq from the same cell lines were downloaded from ENCODE (https://www.encodeproject.org). SREBP1 binding peaks were called with Model-based Analysis of ChIP-Seq (MACS) and then annotated with ChIPseeker R package^103^. We performed *de novo* motif analysis of the SREBP1-binding sites with HOMER software (http://homer.ucsd.edu/homer/). Overlapping genes between RNA-Seq and ChIP-Seq datasets were identified by BioVenn^104^. Pathway annotation network analysis was performed on Cytoscape using ClueGo with REACTOME pathway^105^.

### ChIP quantitative PCR (ChIP-qPCR)

For each ChIP assay, 5 × 10^7^ cells were used. Chromatins from HT-144 cells were fixed with 1% formaldehyde (Polysciences) and prepared with Magna ChIP HiSens Chromatin Immunoprecipitation Kit (EMD Millipore). Nuclei were sonicated on a sonic dismembrator 550 (Thermo Fisher Scientific) with a microtip (model 419) from Misonix Inc. Lysates were sonicated on ice with 10 pulses of 20 sec each (magnitude setting of 3.5) and a 40-sec rest interval. Supernatant was used for immunoprecipitation with the following antibodies: rabbit anti-SREBP1 (H-160, Santa Cruz Biotechnology), rabbit anti-RNA Polymerase II (8WG16, BioLegend), rabbit anti-RNA polymerase II CTD repeat YSPTSPS (phospho S2) antibody (ab5095, Abcam), rabbit anti-RNA polymerase II CTD repeat YSPTSPS (phospho S5) (ab5131, Abcam), rabbit anti-Histone H3 (tri methyl K36) (ab9050, Abcam). qPCR reactions in triplicates were performed on a LightCycler 480 (Roche) using LightCycler 480 SYBR Green I Master (Roche). qPCR primers are listed in Table 2.

**Table 2:** The primers used for ChIP-qPCR assays.

FASN -2797F	5′-ACCTGCAAGGATGAGAAACG-3′
FASN -2797R	5′-AGCATTGTGTTCGTGTGGAG-3′
FASN -2517F	5′-TCAGGAGCCCAGTCTCTGTC-3′
FASN -2517R	5′-TCTGCCCAGTTCAGAAAGGT-3′
FASN -2275F	5′-TTGCACCCCCAGTGTAAAAT-3′
FASN -2275R	5′-CCTTTGTCTGTGTGGTGTGG-3′
FASN -2061F	5′-CACACATGTCCCCTGGAATC-3′
FASN -2061R	5′-AGGAGGCAGAGGTTGCAGT-3′
FASN -1819F	5′-CTGTTTCCCAGGCTTGTCTAC-3′
FASN -1819R	5′-GTTGGAGACTCGTCCAGCAC-3′
FASN -1556F	5′-GAGGTTTGGAGCATGGAGAG-3′
FASN -1556R	5′-AGGGTCAAGGAAGGACAAGG-3′
FASN -1438F	5′-CCCTGTCTTCCCTTGTCCTT–3′
FASN -1438R	5′-CGCTTCCTTCCTCTCCTGA-3′
FASN -1254F	5′-GCTGAGCCCTCAAAGTAGGA-3′
FASN -1254R	5′-CTCCCTTTTCTGACCGCTTC-3′
FASN -977F	5′-CACACCAGGTGGGTGTCC-3′
FASN -977R	5′-CCCCTCCTTAGCAGCTTCC-3′
FASN -160F (SRE)	5′-CACGAGCATCACCCCACC-3′
FASN -160R (SRE)	5′-TATTTAAACCGCGGCCATCC-3′
FASN -13F (TSS)	5′-TAGAGGGAGCCAGAGAGACG-3′
FASN -13R (TSS)	5′-GCTGCTCGTACCTGGTGAG-3′
FASN 184F	5′–CTCCTCATCCTCCGCTCTC-3′
FASN 184R	5′-CATCCGCCCACCTACTCC-3′
FASN 712F	5′-CAGCCAAGCCTCTGTGAATC-3′
FASN 712R	5′-CTGGTCTGGCCACTTGCAC-3′
FASN 959F	5′-CTGTGGTGTGTGGGTTGGTA-3′
FASN 959R	5′-TCCAACCTGGAGAACCACTC-3′
FASN 1523F	5′-GCCAATACCCTGAACTGAATG-3′
FASN 1523R	5′-CTTCCTCATGTGGCCAGTTC-3′
FASN 1687F	5′-CATGGGTGTCCACCTGTTCT-3′
FASN 1687R	5′-TCTCCGACTCTGGCAGCTT-3′
FASN 1984F	5′-CTGCTGTGCTGTGTCCTCAC-3′
FASN 1984R	5′-CTAGGCCACTCTGGTGCAAT-3′
FASN 2217F	5′-CTTGTTACGGAGGAGCCAAG-3′
FASN 2217R	5′-GGAGCACATCTGGTATGCAA-3′
FASN 2430F	5′-CAAGCTGTGAGTCAGCATGG-3′
FASN 2430R	5′-ACGACCAGATCTGCTGCAC-3′
FASN 2716F	5′-GCAGCTTCCGTCAGAGATTT-3′
FASN 2716R	5′-CGAAGAAGGAGGCATCAAAC-3′
FASN 3802F	5′-TTGGGTGTGAGCGGTTCA-3′
FASN 3802R	5′-AACCTTAGTCTTGGGGTGGG-3′
FASN 4814F	5′-CAGCATCGCACTGGACAC-3′
FASN 4814R	5′-ATCCCCAGCCTCAAGAACTG-3′
FASN 5845F	5′-ACTGCTGCTTTCTCTTTGCC-3′
FASN 5845R	5′-TGTCAGCCTCAGTGCCTC-3′
FASN 6862F	5′-TTTCCTGAGCATGCTGAACG-3′
FASN 6862R	5′-CACCTGCTGCACCTCTGG-3′
FASN 7800F	5′-AGGGGACAGTGCATCAAAGA-3′
FASN 7800R	5′-GCCACTCTATCCTACGGGG-3′
FASN 8819F	5′-CTTATCCAGGCTCCCACGAT-3′
FASN 8819R	5′-GTCCACCTTCTTGAGCTCCT-3′
FASN 9819F	5′-CAGCCGCCATCTACAACATC-3′
FASN 9819R	5′-GAGACTCGGAGCTGGTGTC-3′
FASN 10834F	5′-TGTCACCGCCATCCACAT-3′
FASN 10834R	5′-GTGAGTGCGGCATACTTGG-3′
FASN 11864F	5′-CAAGCTGCCAGAGGACCC-3′
FASN 11864R	5′-CACCTCCACCACCTTCATCT-3′
FASN 12881F	5′-AAGTCCTTCTACGGCTCCAC-3′
FASN 12881R	5′-CCTTCAGAGACTCCACCCAG-3′
FASN 13867F	5′-CCTGCTAGGTATGGAGTTCTCG–3′
FASN 13867R	5′-CGGTGACAGCAGGACAGA-3′
FASN 14857F	5′-TTCTTCAACGAGAGCAGTGC-3′
FASN 14857R	5′-GGAACACCGTGCACTTGAG-3′
FASN 15818F	5′-TTGAGAGATGGCTTGCTGGA-3′
FASN 15818R	5′-GGCTGTCAACAAACCACCTT-3′
FASN 16820F	5′-CATACAGGCCGTCCCCATG-3′
FASN 16820R	5′-AGCACCAGGTTGAGCTCAC-3′
FASN 17838F	5′-CTACGTACTGGCCTACACCC-3′
FASN 17838R	5′-CTCTGAGAGGAAGGAGGGAC-3′
FASN 19809F (TTS)	5′-GTCTGGACCCCGTTTCATTT-3′
FASN 19809R (TTS)	5′-CAGGGAGGAGGGAAGAAAGG-3′
SCD -152F (SRE)	5′-CAGCCCCTTCCAGAGAGAAA-3′
SCD -152R (SRE)	5′-CGAAGGGAATTTGGTGCCAC-3′
SCD 12F (TSS)	5′-GTGGCACCAAATTCCCTTCG-3′
SCD 12R (TSS)	5′-GACACCGACACCACACCA-3′
SCD 245F	5′-CTTGGCAGCGGATAAAAGGG-3′
SCD 245R	5′-GCACGCTAGCTGGTTGTC-3′
SCD 404F	5′-CTTCGAAACCGCAGTCCTC-3′
SCD 404R	5′-CAGGCTGGGAAACTCACATC-3′
SCD 651F	5′-CCGTGAGTTGGGAATGTGGA-3′
SCD 651R	5′-TCACATCCCCACGAAGACAA-3′
SCD 883F	5′-GGAACTTTTCTCCGTTGCGT-3′
SCD 883R	5′-GCCACTCCGCTCTCTAATCA-3′
SCD 1006F	5′-CCACGTGTCTCTTCTCCTGA-3′
SCD 1006R	5′-TTCCAAGTAGAGGGGCATCG-3′

### Cell viability assay and synergy score

For dose-response assay, 1,000 - 2,000 cells were seeded per well in Falcon 96-Well Imaging Microplate with Lid (Corning). Cells were then cultured in the absence or presence of indicated drugs for 4–6 days. Relative cell viability was quantified with the CellTiter-Glo Luminescent Cell Viability Assay Kit (G7572, Promega) and luminescent signals were measured with the Envision 2103 Multilabel Microplate Reader (Perkin Elmer). Experiments were performed independently for two or three times. Titration curves and IC50 values were generated using GraphPad Prism software. Dose-response data of drug combinations were analyzed with the SynergyFinder R package for synergism^70^. Synergy scores were calculated across all concentration combinations using Loewe or Bliss models.

## Supplementary information

Supplementary information

## Data Availability

Scripts used to perform the bioinformatics analyses for this study is available on GitHub https://github.com/a3609640/DNFA-gene-analysis.
