# Supplementary material for: SREBP1-dependent de novo fatty acid synthesis gene expression is elevated in malignant melanoma and represents a cellular survival trait
Source: Sci Rep. 2019 Jul 17;9:10369. doi: 10.1038/s41598-019-46594-x (PMC6637239; doi:10.1038/s41598-019-46594-x)
Supplement: Supplementary file 1 — Supplementary information [file 41598_2019_46594_MOESM1_ESM.pdf]

SREBP1-dependent *de novo* fatty acid synthesis gene expression is elevated in malignant melanoma and represents a cellular survival trait

Su Wu[1,2,*,#] and **Anders M. Näär**[1,2,*,#]

[1]Massachusetts General Hospital Center for Cancer Research, Charlestown, MA, USA;
[2]Department of Cell Biology, Harvard Medical School, Boston, MA, USA

*Correspondence: Anders M Näär, naar@berkeley.edu; Su Wu, Su_Wu@hms.harvard.edu

[#]Current Address: S.W.: Department of Biological Chemistry and Molecular Pharmacology, Harvard Medical School, Boston, MA, USA

[#]Current Address: A.M.N.: Department of Nutritional Sciences & Toxicology, University of California, Berkeley, Berkeley, CA, USA

TABLE OF CONTENTS

Supplementary Table **3**

Supplementary Table 1: Correlation between mRNA and protein levels of lipogenic enzymes in cancer. 3

Supplementary Figures and Legends **4**

Supplementary Figure 1: Elevated expression of *ACLY* and *ACSS2* genes is prevalent in many cancers, especially in melanomas, and has prognostic value. 4

Supplementary Figure 2: Elevated expression of DNFA genes has prognostic value. 5

Supplementary Figure 3: Expression of *HMGCS1* and *HMGCR* genes in cancers. 6

Supplementary Figure 4: DNFA gene expression in melanomas vs. normal skin tissues. 7

Supplementary Figure 5: DNFA gene expression in malignant melanomas vs. benign nevi. 8

Supplementary Figure 6: DNFA gene expression has no significant correlation with common oncogenic driver mutations in skin cutaneous melanoma (SKCM). 9

Supplementary Figure 7: SREBP1 regulates elevated DNFA gene expression in melanoma cells. 10

Supplementary Figure 8: SREBP1 consistently regulates DNFA genes across multiple cell lines. 11

Supplementary Figure 9: SREBP1's most profound and direct effects are on DNFA pathway genes. 12

Supplementary Figure 10: SREBP1 predominantly binds to the TSS sites of DNFA gene promoters. 13

Supplementary Figure 11: SREBP1-binding associates with productive transcription elongation of RNAP II on DNFA genes. 14

Supplementary Figure 12: DNFA gene expression after vemurafenib treatment in HT-144 cells. 15

Supplementary Figure 13: DNFA gene expression after dabrafenib treatment in HT-144 cells. 16

Supplementary Figure 14: DNFA gene expression after BRAF inhibitor (BRAFi) and ERK inhibitor (ERKi) treatment in A375 cells. 17

Supplementary Table 1 | Correlation between mRNA and protein levels of lipogenic enzymes in cancer. Pearson correlation of RNA-Seq to protein abundance for DNFA and DNCS genes across 77 breast cancer samples in CPTAC. All p-values were Benjamini-Hochberg corrected. These data are directly excerpted from Supplementary Table 9 of Mertins et al. (2016).

Gene ID	RefSeq Protein ID	Pearson correlation	p-value	FDR p-value
ACSS2	NP_001070020	0.6902	1.40E-12	1.60E-11
ACLY	NP_942127	0.7759	< 2e-16	< 2e-16
ACACA	NP_942133	0.7374	6.20E-15	1.40E-13
FASN	NP_004095	0.8516	< 2e-16	< 2e-16
SCD	NP_005054	0.527	1.70E-06	5.30E-06
ACSL1	NP_001986	0.6448	1.10E-10	8.10E-10
HMGCS1	NP_001091742	0.7948	< 2e-16	< 2e-16
HMGCR	NP_000850	-0.014	0.90849	0.92469
MVD	NP_002452	0.8084	< 2e-16	< 2e-16
MVK	NP_000422	0.6456	1.00E-10	7.60E-10
PMVK	NP_006547	0.5238	6.20E-07	2.10E-06
SREBF1	NP_001005291	0.0283	0.87765	0.8997
SREBF2	NP_004590	0.0896	0.46051	0.52787

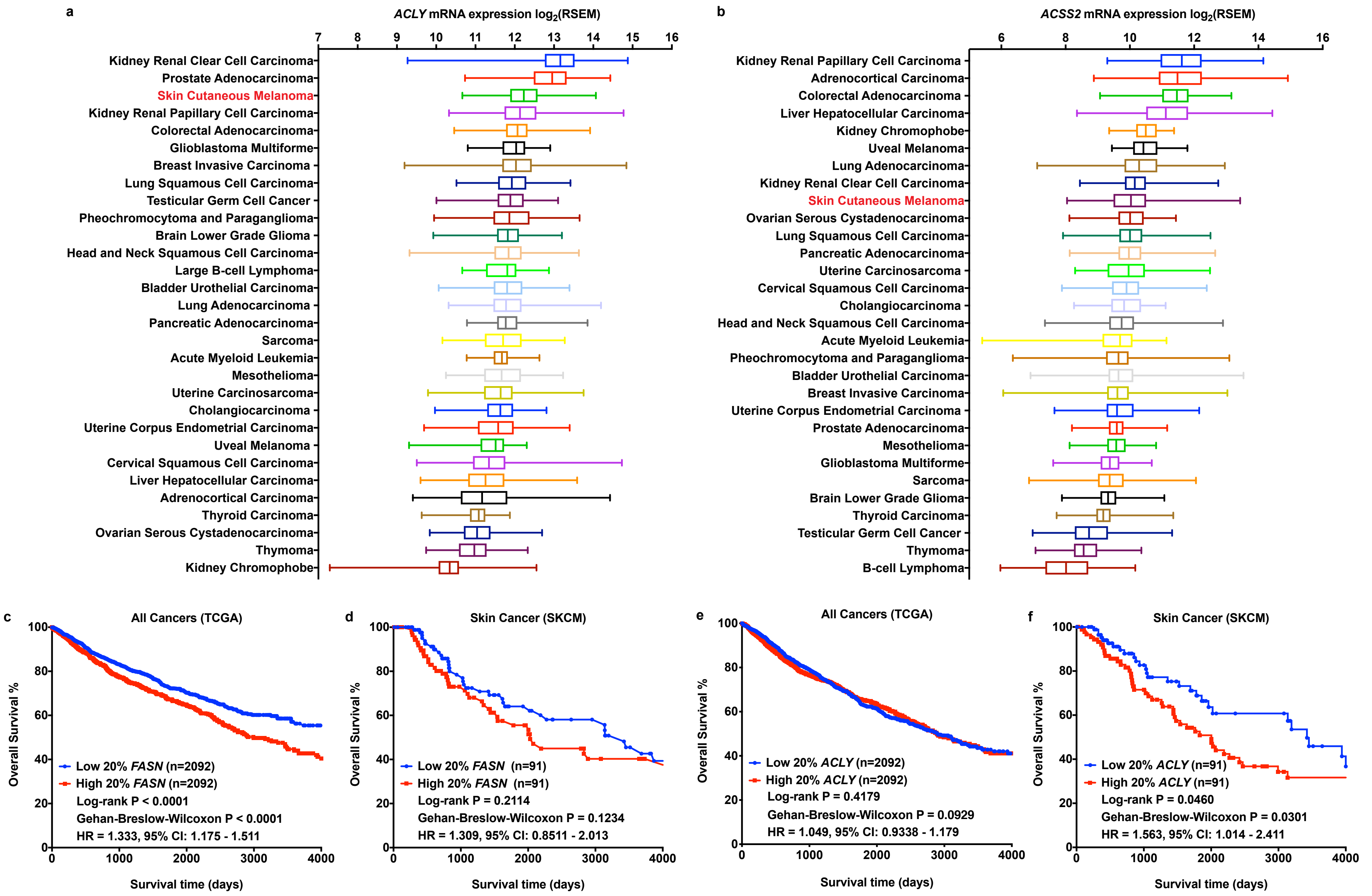

Supplementary Figure 1 | Elevated expression of *ACLY* and *ACSS2* genes is prevalent in many cancers, especially in melanomas, and has prognostic value. a, b, Expression of *ACLY* and *ACSS2* genes is compared using RSEM normalized RNA-Seq data from 10,460 tumor samples in The Cancer Genome Atlas (TCGA). The box and whiskers plots represent gene expression in 30 TCGA cancer types. **c-f,** Correlation between *FASN*, *ACLY* expression and prognosis in all cancer patients from TCGA dataset (**c, e**) and in melanoma patients from SKCM dataset (**d, f**). Differences in overall survival rates are computed between patients with top 20% RNA-Seq counts of DNFA genes in their tumor samples and those with bottom 20% expression, as the Kaplan-Meier plots.

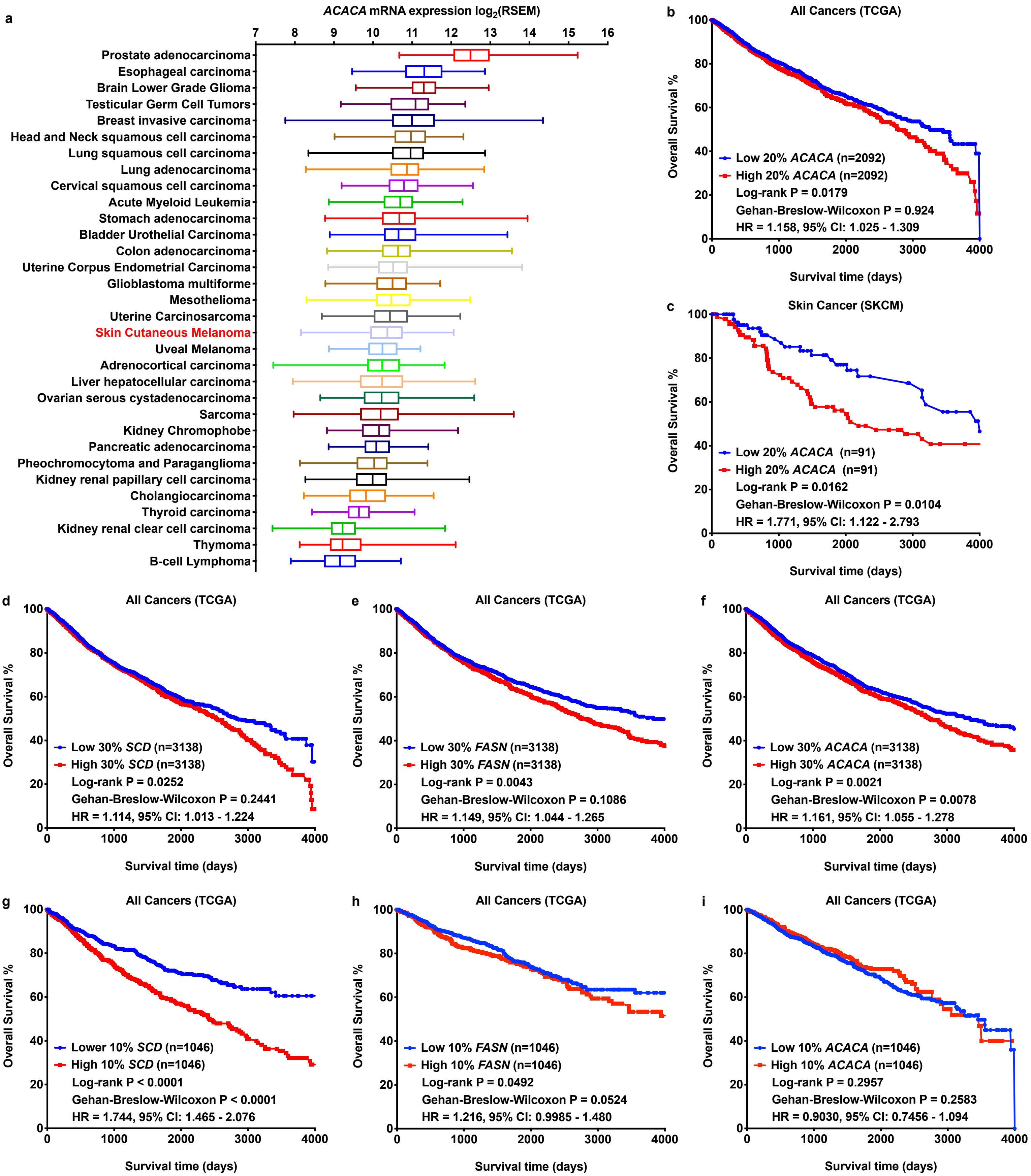

Supplementary Figure 2 | Elevated expression of DNFA genes has prognostic value. a, Expression of *ACACA* gene is compared using RSEM normalized RNA-Seq data from all tumor groups in The Cancer Genome Atlas (TCGA), as the box and whiskers plot. Correlation between *ACACA* expression and prognosis in all cancer patients from TCGA dataset (**b**) and melanoma patients from SKCM dataset (**c**). **d-f,** Differences in overall survival rates are computed from all TCGA patients with top 30% DNFA RNA-Seq counts in their tumor samples and those with bottom 30% expression, as the Kaplan-Meier plots. **g-i,** Differences in overall survival rates were computed with 10% cutoff for DNFA expression, as the Kaplan-Meier plots.

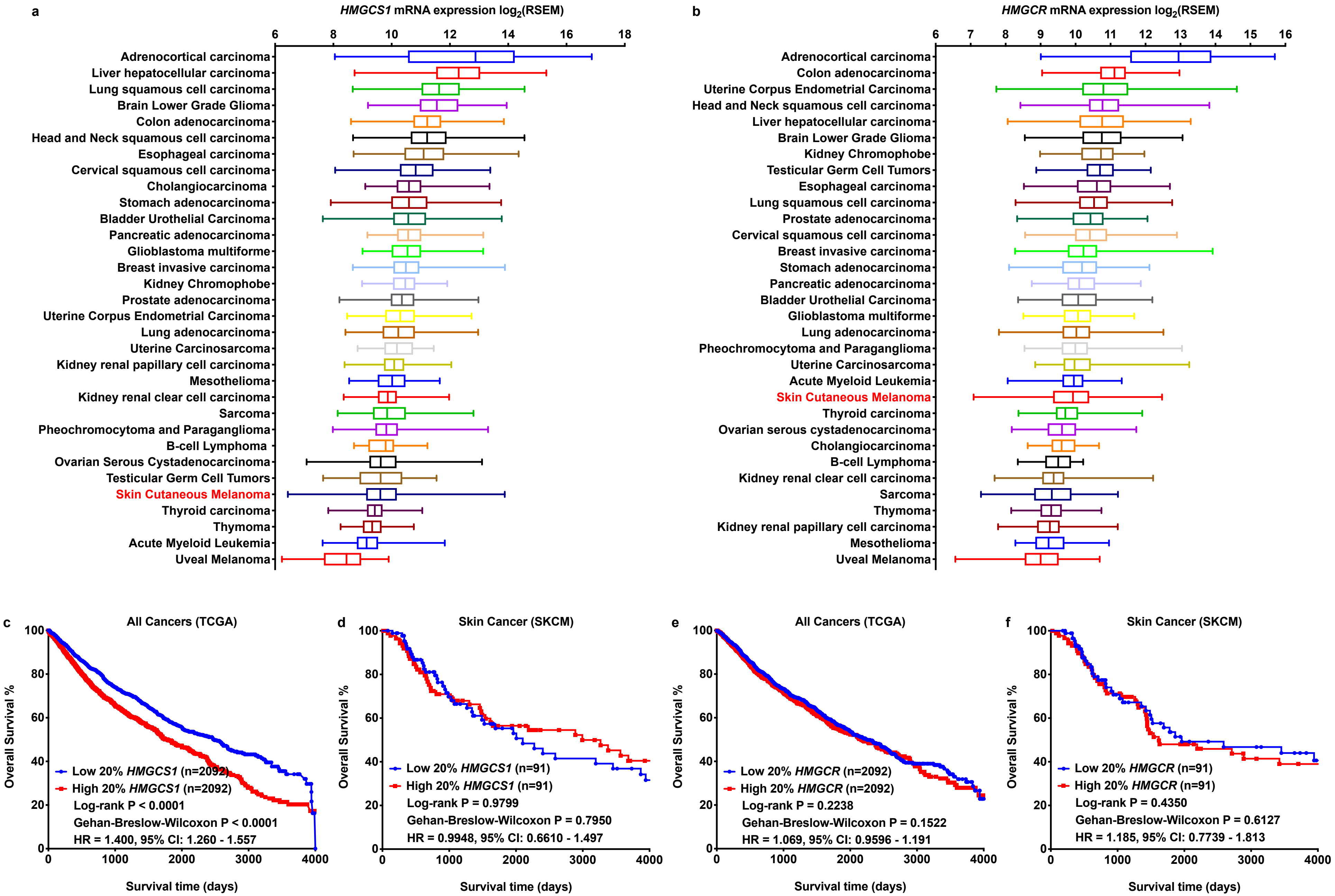

Supplementary Figure 3 | Expression of *HMGCS1* and *HMGCR* genes in cancers. a, b, Expression of *HMGCS1* and *HMGCR* genes is compared using RSEM normalized RNA-Seq data from all tumor groups in The Cancer Genome Atlas (TCGA), as the box and whiskers plots. **c-f,** Correlation between *HMGCS1*, *HMGCR* expression and prognosis in all cancer patients from TCGA dataset (**c, e**) and in melanoma patients from SKCM dataset (**d, f**). Differences in overall survival rates are computed between patients with top 20% RNA-Seq counts of DNFA genes in their tumor samples and those with bottom 20% expression, as the Kaplan-Meier plots.

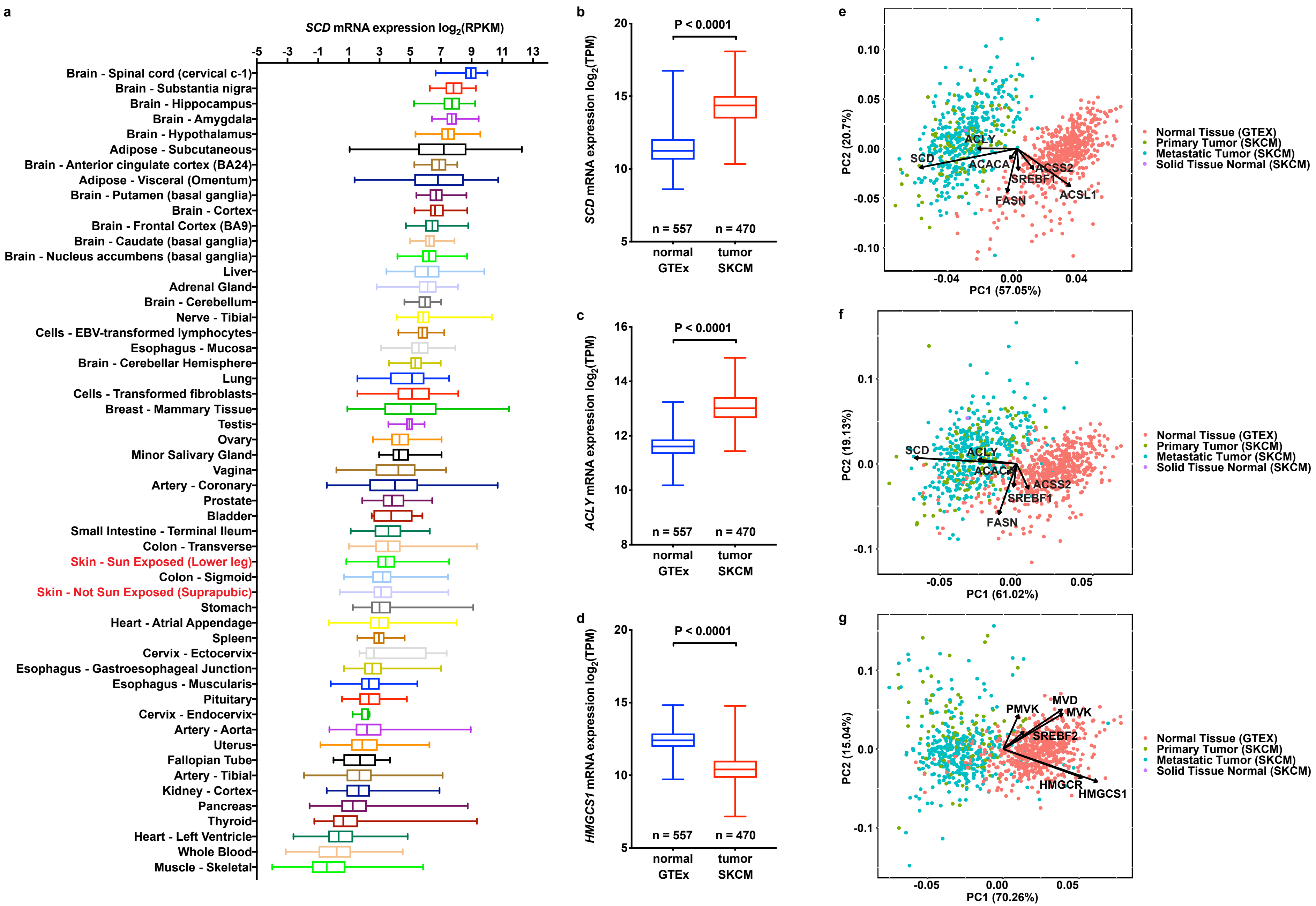

Supplementary Figure 4 | DNFA gene expression in melanomas vs. normal skin tissues. a, *SCD* gene expression was compared across different normal tissue types using RNA-Seq data downloaded from Genotype-Tissue Expression (GTEx). **b-d,** expression of *SCD*, *ACLY* and *HMGCS1* was compared between normal skin tissues (both Sun Exposed and Not Sun Exposed skin tissues from GTEx) and skin tumors. RNA-Seq data of normal samples from GTEx and RNA-Seq data of skin tumor samples from TCGA were normalized by TOIL and DESeq2 methods for cross-study comparison by UCSC Xena. P values were calculated by the non-parametric Mann Whitney test. **e-g,** RNA-Seq data of DNFA and DNCS genes separate TCGA tumor samples from GTEx normal tissue samples by principal component analysis (PCA). Arrow lines indicate the contribution of each gene to the separation between tumor and normal samples.

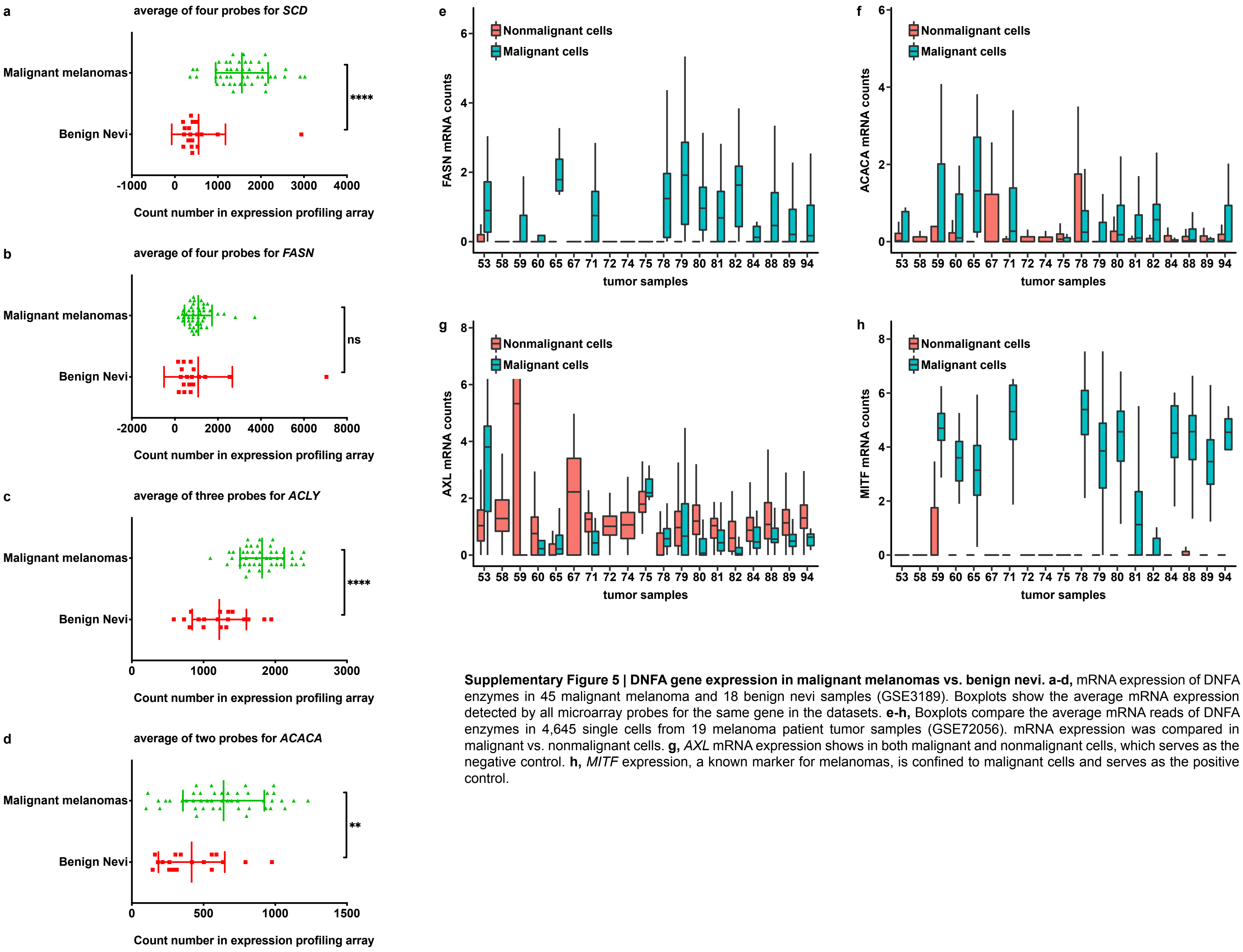

Supplementary Figure 5 | DNFA gene expression in malignant melanomas vs. benign nevi. a-d, mRNA expression of DNFA enzymes in 45 malignant melanoma and 18 benign nevi samples (GSE3189). Boxplots show the average mRNA expression detected by all microarray probes for the same gene in the datasets. **e-h,** Boxplots compare the average mRNA reads of DNFA enzymes in 4,645 single cells from 19 melanoma patient tumor samples (GSE72056). mRNA expression was compared in malignant vs. nonmalignant cells. **g,** *AXL* mRNA expression shows in both malignant and nonmalignant cells, which serves as the negative control. **h,** *MITF* expression, a known marker for melanomas, is confined to malignant cells and serves as the positive control.

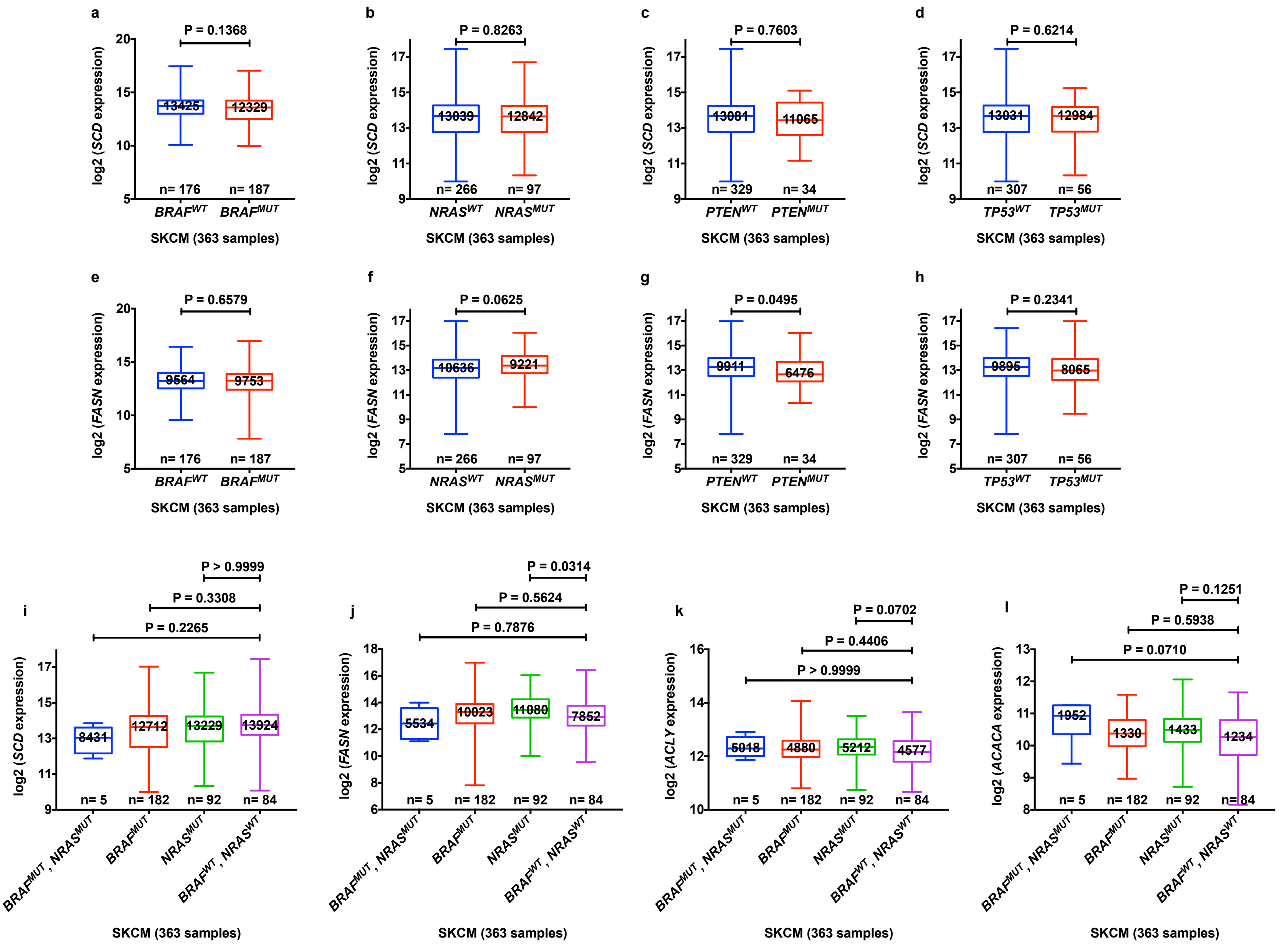

Supplementary Figure 6 | DNFA gene expression has no significant correlation with common oncogenic driver mutations in skin cutaneous melanoma (SKCM). Boxplots (**a-h**) compare the RNA-Seq counts of *SCD* and *FASN* in TCGA skin cancer samples (SKCM), in which the indicated gene is found to be wild-type or mutated. Box plots (**i-l**) compare the mRNA abundance of *SCD* and *FASN* in skin cancer samples with *BRAF*/*NRAS* mutation (single mutation or double mutations). Data are from the TCGA SKCM study group. P values were calculated using the non-parametric Mann Whitney test.

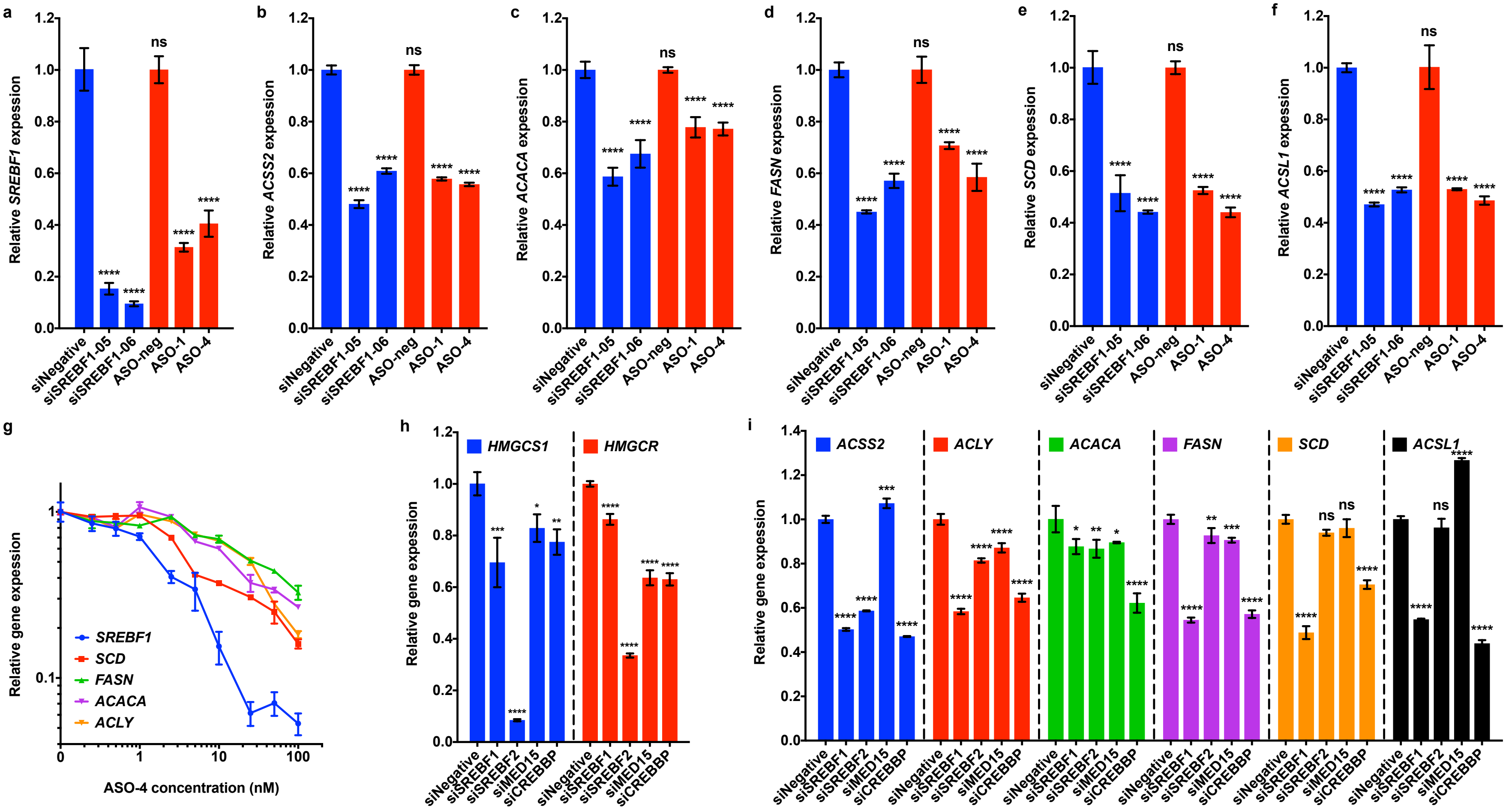

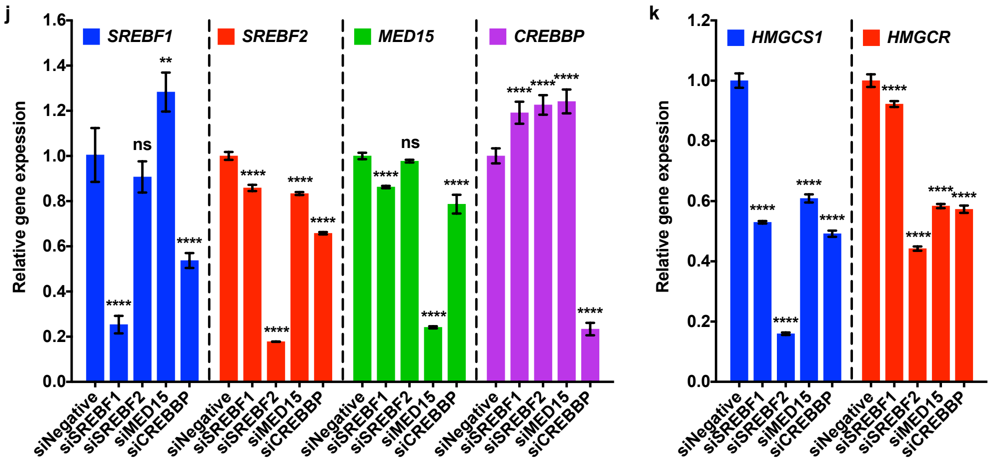

Supplementary Figure 7 | SREBP1 regulates elevated DNFA gene expression in melanoma cells. a-f, HT-144 cells were transfected with single siRNA reagent (50 nM) or ASOs (5 nM) for SREBF1 in 1% ITS medium and assayed with RT-qPCR three days later to compare expression of DNFA enzymes from treatment groups relative to that from negative control treatment group. **g,** HT-144 cells were transfected with various doses of ASO-4 in 10% FBS medium. mRNAs were harvested 48 hours after transfection and analyzed by RT-qPCR to examine relative expression of the indicated genes to mock. **h,** RT-qPCR assay of mRNA shows relative expression of cholesterol biosynthesis enzymes from siRNAs treatment groups to that of siNegative treatment in HT-144 cells (as the supplementary data to Fig. 2d-e). **i-k,** A375 cells were transfected with pooled siRNAs (50 nM) in 1% ITS medium for three days to deplete *SREBF1*, *SREBF2*, *MED15* or *CREBBP*. RT-qPCR assay of mRNA shows expression of DNFA genes from siRNAs treatment groups relative to expression under siNegative treatment.

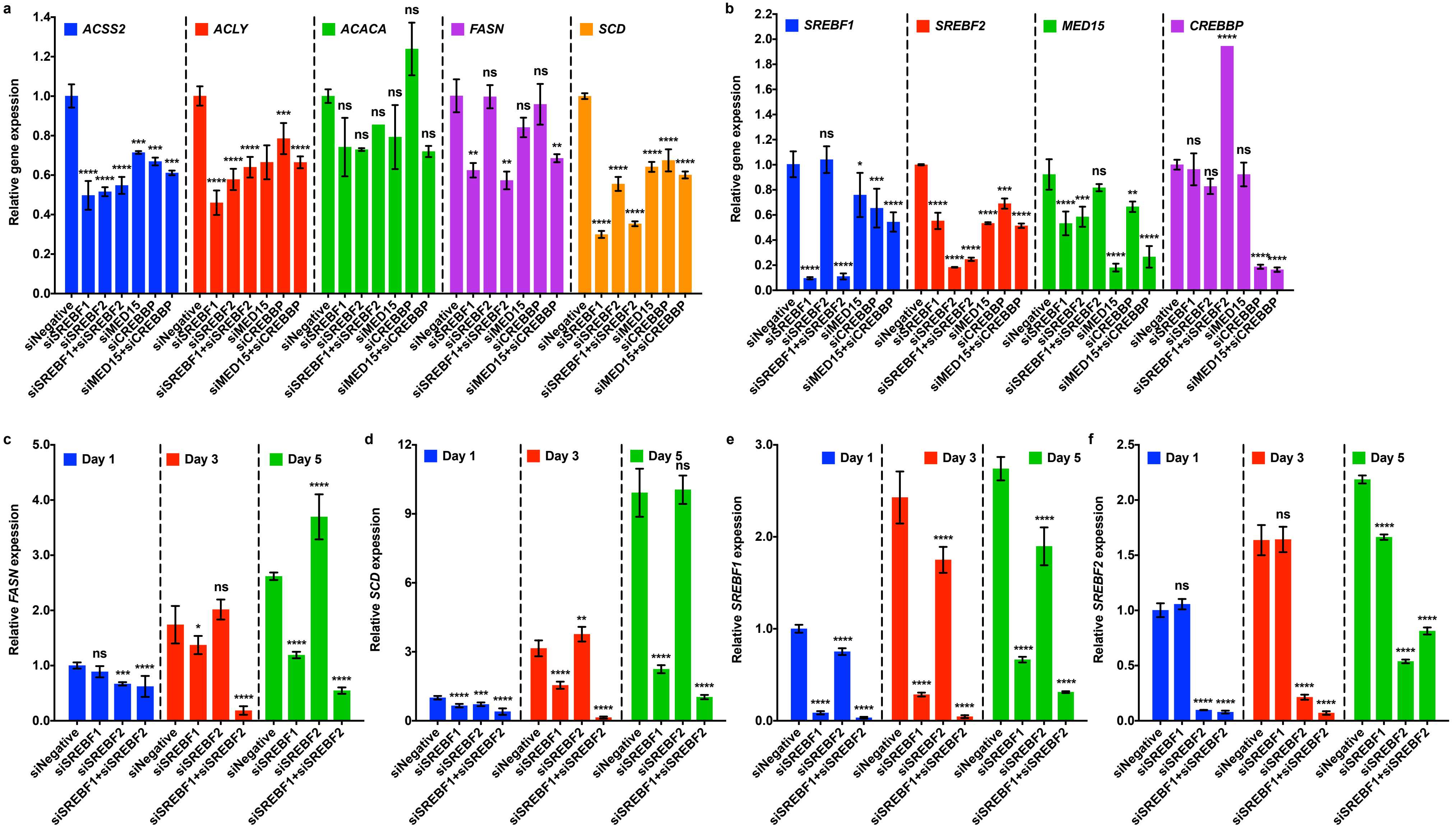

Supplementary Figure 8 | SREBP1 consistently regulates DNFA genes across multiple cell lines. a-b, MEL-JUSO cells were transfected with pooled siRNAs (50 nM) to deplete *SREBF1*, *SREBF2*, *MED15*, *CREBBP* or in combination in 1% ITS medium. DNFA gene expression was assayed by RT-qPCR analysis three days after transfection. Data are presented as mean ± SD and quantified from triplicates. One-way ANOVA tests were performed. **c-f,** A375 cells were transfected with pooled siRNAs designed to deplete *SREBF1* or *SREBF2* or both in 1% ITS medium for time course RT-qPCR assay. RT-qPCR assay of mRNA shows expression of DNFA enzymes from siRNAs treatment groups relative to expression under siNegative treatment at day 1 (normalized as 1). Data are presented as mean ± SD and quantified from triplicates. One-way ANOVA tests were performed. ns, not significant; *, $P < 0.05$; **, $P < 0.01$; ***, $P < 0.001$; ****, $P < 0.0001$.

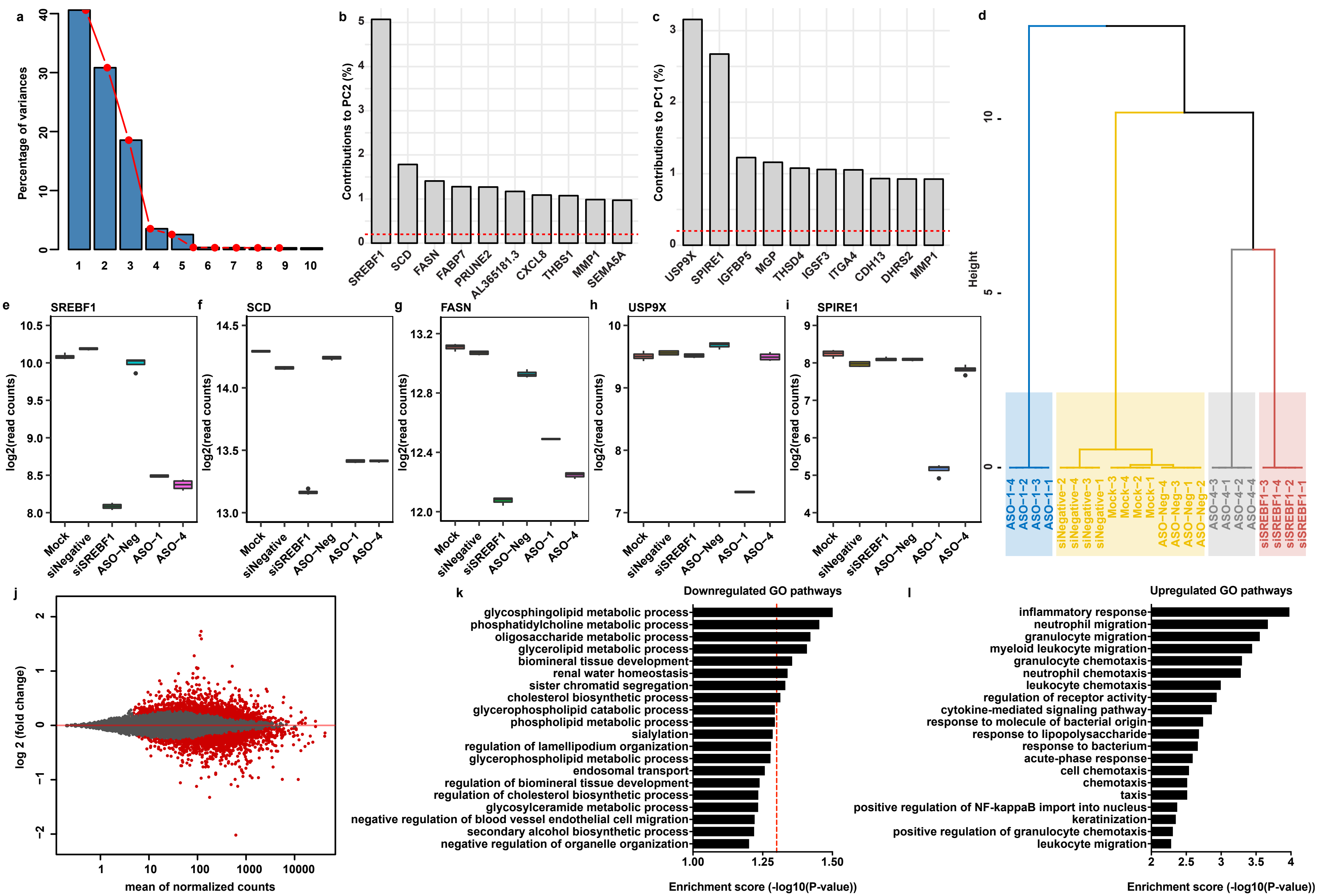

Supplementary Figure 9 | SREBP1's most profound and direct effects are on DNFA pathway genes. a, the bar graph shows the variance retained by each principal component (PC) in Figure 3a. **b-c,** the top ten genes mainly contributed to PC1 and PC2 in Fig. 3a. The contributions of variables in accounting for the variability in a given principal component are (in percentage): (variable cos2 *100) / (total cos2 of the component). The red dashed line on the graph above indicates the expected average contribution from a gene. **d,** hierarchical clustering was performed on RNA-Seq data for identifying groups of similar observations (RNA-Seq data of each group were represented as four technical replicates). **e-g,** the decrease of RNA read counts for *SREBF1* and its targeted genes in RNA-Seq data are presented as boxplots. **h-i,** off-target genes affected by ASO-1. RNA-Seq data show *SPIRE1* and *USP9X* genes are downregulated only by ASO-1. **j,** The MA plot shows the differences of RNA-Seq data between siSREBF1 and siNegative by transforming the data onto log2 ratio (with LFC shrinkage) and mean average RNA counts. Points were colored red when the adjusted p value is less than 0.1. **k, l,** top 20 enriched biological process in Gene Ontology (GO) pathways were discovered from differentially expressed genes found in RNA-Seq analysis (siSREBF1 vs siNegative group) using DESeq2 and Generally Applicable Gene-set Enrichment (GAGE) packages. Red dash line marks P value = 0.05.

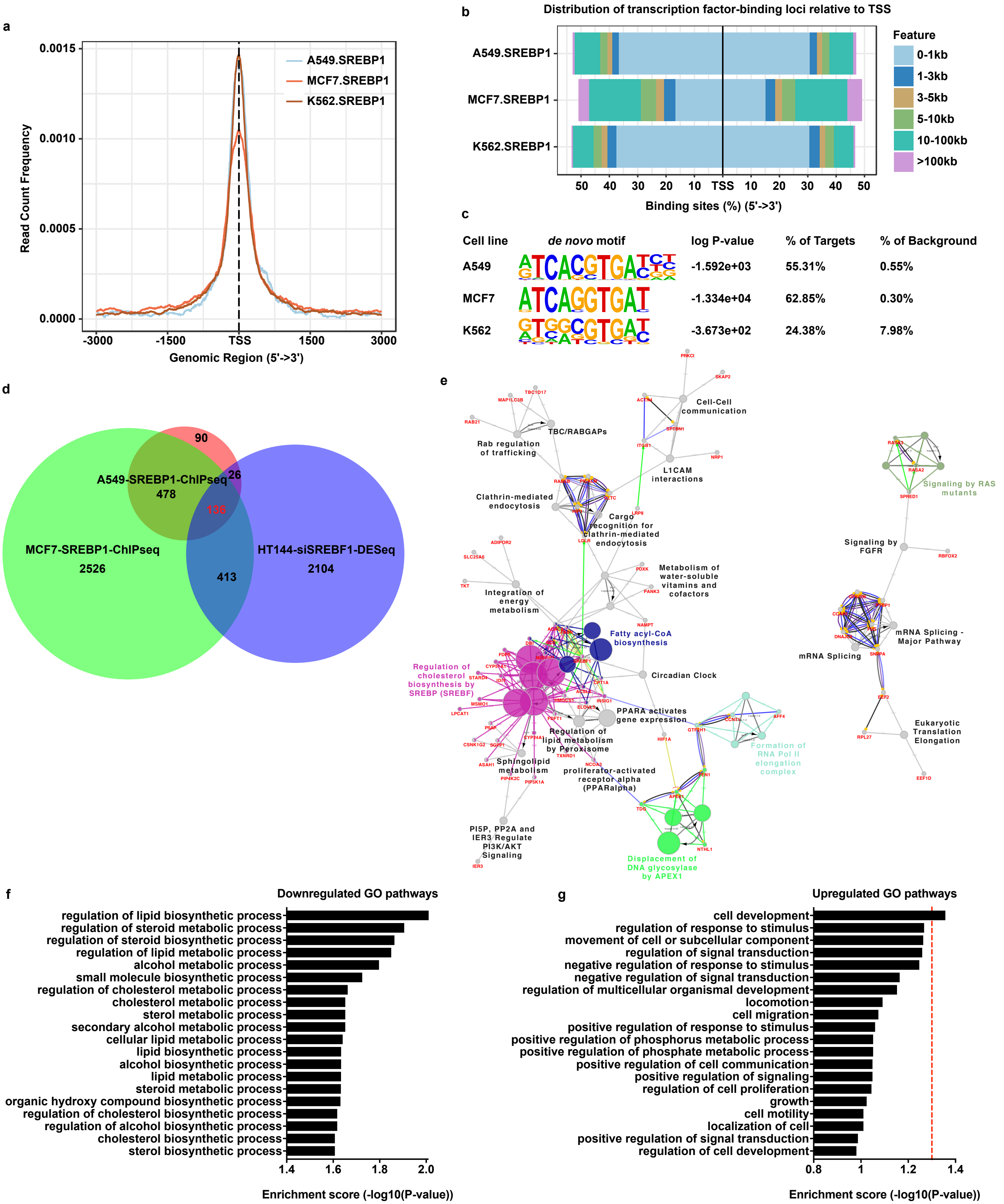

Supplementary Figure 10 | SREBP1 predominantly binds to the TSS sites of DNFA gene promoters. a, b, public SREBP1 ChIP-Seq data from three cell lines were analyzed for peak distribution by MACS. These cells lines were selected because they are all the cancer cell lines in the Encyclopedia of DNA Elements (ENCODE) database with SREBP1 ChIP-Seq results that are free of the warning "insufficient read length". **c,** *de novo* motif discovery (HOMER) on public SREBP1 ChIP-Seq data, using input ChIP-Seq data from the same cell lines as control. **d,** Venn diagram shows the 136 overlapping genes (in red) from SREBP1 ChIP-Seq in MCF7, A549 cells and differential expressed genes from RNA-Seq in HT-144 cells (siSREBF1 vs siNegative, P value < 0.05). **e.** Reactome pathway analysis on 136 overlapping genes from d. **f-g,** top 20 enriched biological process in Gene Ontology (GO) pathways were discovered from overlapping genes in d, using DESeq2 and Generally Applicable Gene-set Enrichment (GAGE) packages. Red dash line marks P value = 0.05.

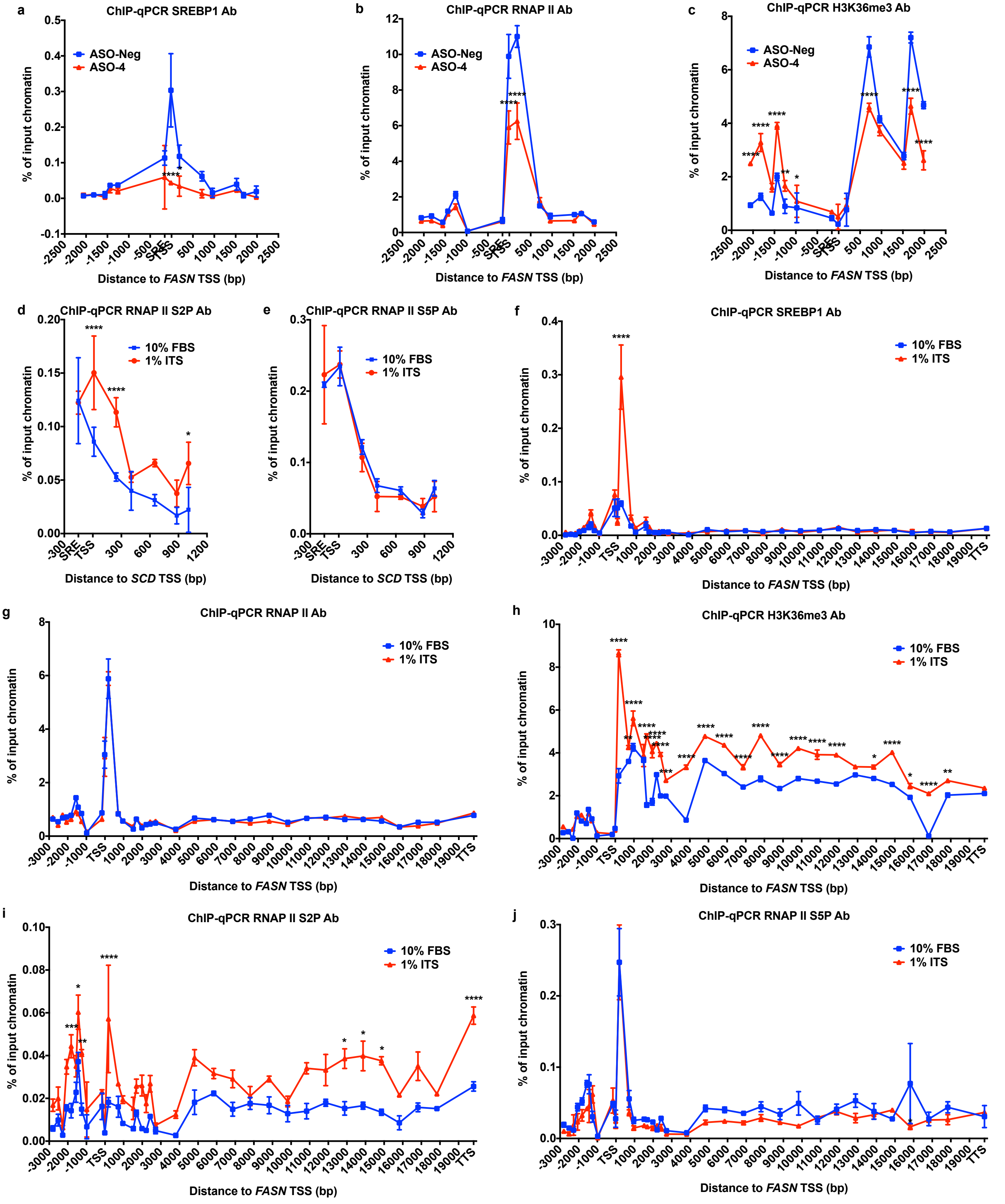

Supplementary Figure 11 | SREBP1-binding associates with productive transcription elongation of RNAP II on DNFA genes. a-c, HT-144 cells were transfected with ASO-4 (5 nM) or control ASO (5 nM), cultured in 1% ITS medium for two days and assayed with ChIP-qPCR assay. ChIP-qPCR signals, shown as percentage of input DNA, for the indicated antibodies at the 5' promoter region of *FASN*. **d-e,** ChIP-qPCR analyses detected protein/DNA bindings with the indicated antibodies at *SCD* promoter in HT-144 cells. **f-j,** ChIP-qPCR analyses detected protein/DNA bindings with the indicated antibodies at *FASN* in HT-144 cells. ChIP-qPCR signals were compared between cells cultured in 10% FBS and 1% ITS medium condition. Data were presented as mean ± SD and quantified from triplicates. Two-way ANOVA tests were performed. *, $P < 0.05$; **, $P < 0.01$; ***, $P < 0.001$; ****, $P < 0.0001$.

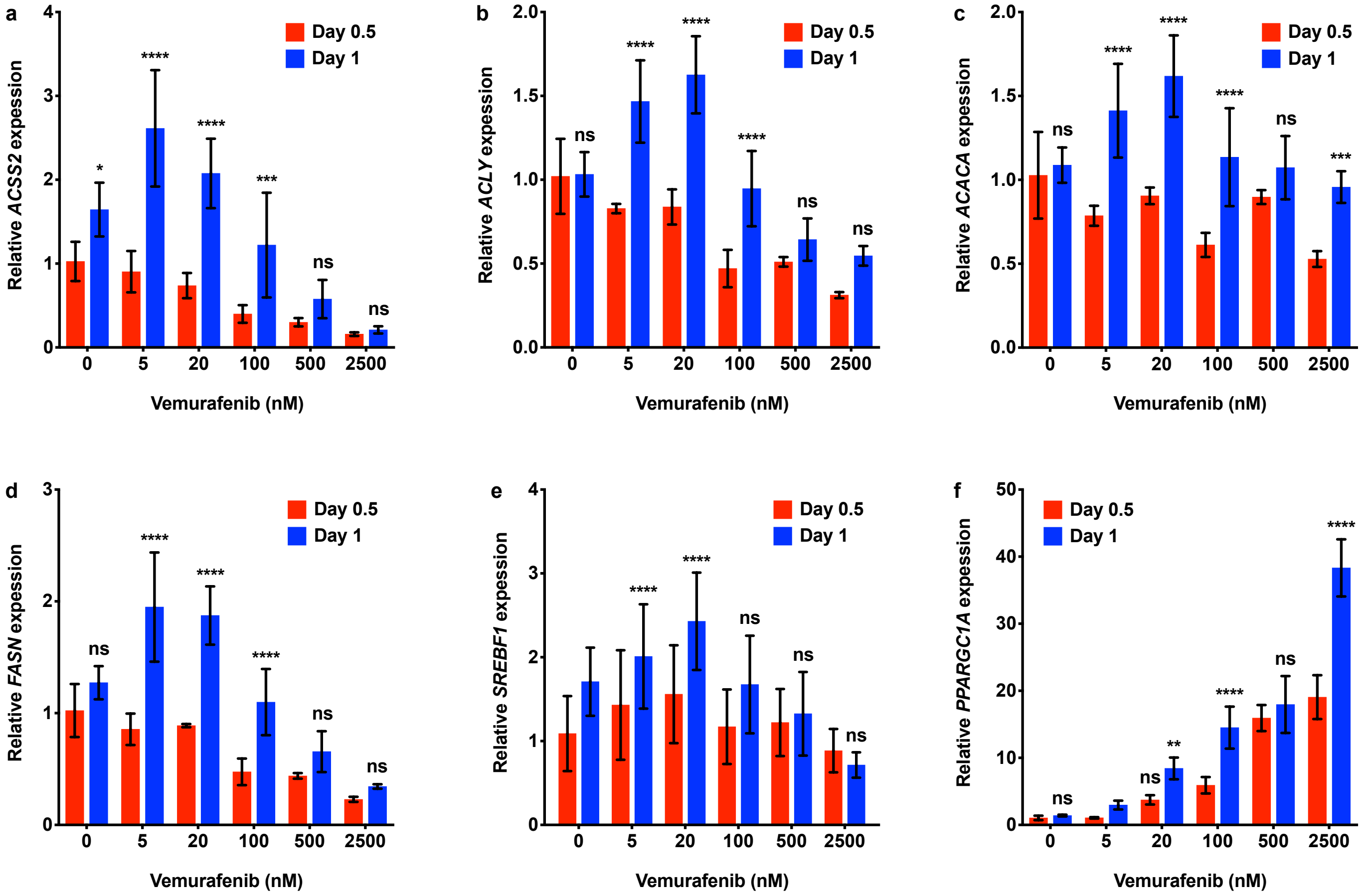

Supplementary Figure 12 | DNFA gene expression after vemurafenib treatment in HT-144 cells. a-f, HT-144 cells were treated with different dosages of vemurafenib for 0.5 or 1 day in 1% ITS medium. DNFA gene expression was assayed by RT-qPCR analysis. Expression of DNFA enzymes from all treatment groups was normalized to expression under DMSO treatment at day 0.5 (normalized as 1). Relative gene expression was compared between 0.5-day and 1-day treatment groups. Data were presented as mean ± SD and quantified from triplicates. Two-way ANOVA tests were performed. *, $P < 0.05$; **, $P < 0.01$; ***, $P < 0.001$; ****, $P < 0.0001$.

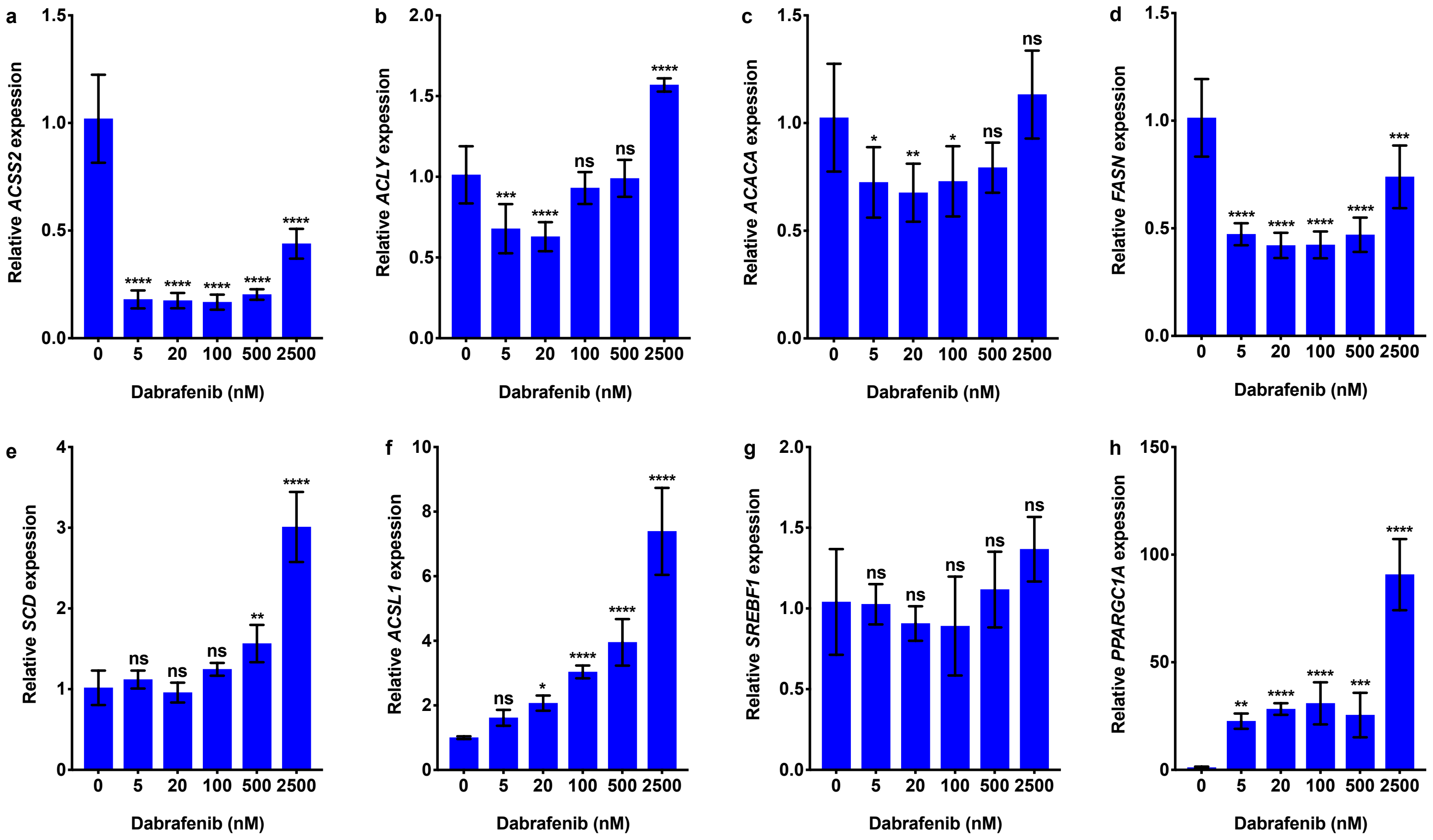

Supplementary Figure 13 | DNFA gene expression after dabrafenib treatment in HT-4 cells. a-h, HT-144 cells were treated with different dosages of dabrafenib for 1 day in 1% ITS medium. DNFA gene expression was assayed by RT-qPCR analysis. Expression of DNFA enzymes from all treatment groups was normalized to expression under DMSO treatment at day one (normalized as 1). Relative gene expression was compared between DSMO and dabrafenib treatment groups. Data were presented as mean ± SD and quantified from triplicates. One-way ANOVA tests were performed. *, $P < 0.05$; **, $P < 0.01$; ***, $P < 0.001$; ****, $P < 0.0001$.

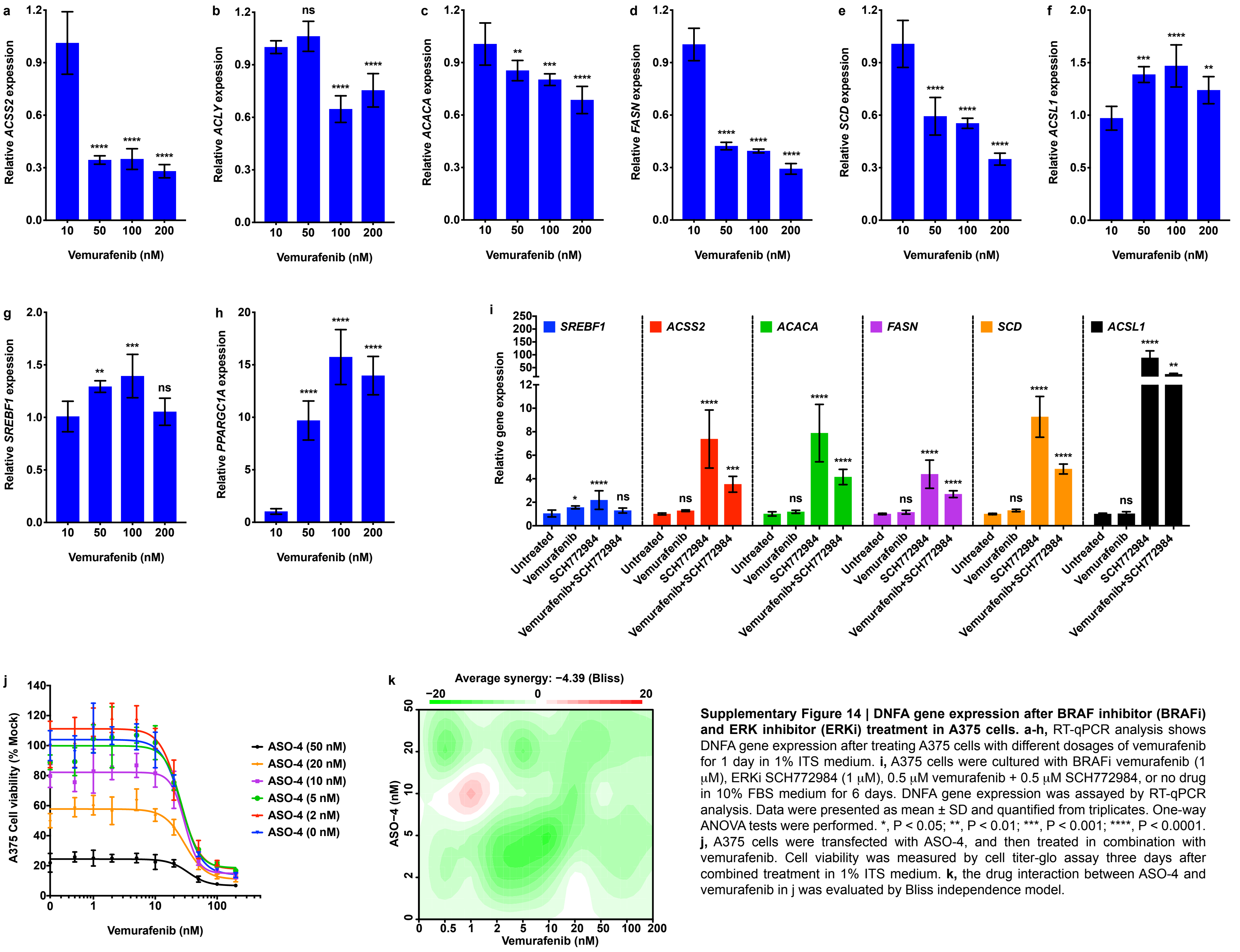

Supplementary Figure 14 | DNFA gene expression after BRAF inhibitor (BRAFi) and ERK inhibitor (ERKi) treatment in A375 cells. a-h, RT-qPCR analysis shows DNFA gene expression after treating A375 cells with different dosages of vemurafenib for 1 day in 1% ITS medium. **i,** A375 cells were cultured with BRAFi vemurafenib (1 μM), ERKi SCH772984 (1 μM), 0.5 μM vemurafenib + 0.5 μM SCH772984, or no drug in 10% FBS medium for 6 days. DNFA gene expression was assayed by RT-qPCR analysis. Data were presented as mean ± SD and quantified from triplicates. One-way ANOVA tests were performed. *, $P < 0.05$; **, $P < 0.01$; ***, $P < 0.001$; ****, $P < 0.0001$. **j,** A375 cells were transfected with ASO-4, and then treated in combination with vemurafenib. Cell viability was measured by cell titer-glo assay three days after combined treatment in 1% ITS medium. **k,** the drug interaction between ASO-4 and vemurafenib in j was evaluated by Bliss independence model.
